# Geographic patterns and environmental factors associated with human yellow fever presence in the Americas

**DOI:** 10.1371/journal.pntd.0005897

**Published:** 2017-09-08

**Authors:** Patricia Najera Hamrick, Sylvain Aldighieri, Gustavo Machado, Deise Galan Leonel, Luz Maria Vilca, Sonia Uriona, Maria Cristina Schneider

**Affiliations:** 1 PAHO Health Emergencies Department, Pan American Health Organization, Washington D.C., United States of America; 2 Veterinary Population Medicine Department, University of Minnesota, Saint Paul, Minnesota, United States of America; 3 Preventive Medicine and Epidemiology Department, Hospital Universitari Vall d’Hebron, Universitat Autonoma de Barcelona, Barcelona, Spain; University of California, Davis, UNITED STATES

## Abstract

**Background:**

In the Americas, yellow fever virus transmission is a latent threat due to the proximity between urban and wild environments. Although yellow fever has nearly vanished from North and Central America, there are still 13 countries in the Americas considered endemic by the World Health Organization. Human cases usually occur as a result of the exposure to sylvatic yellow fever in tropical forested environments; but urban outbreaks reported during the last decade demonstrate that the risk in this environment still exists. The objective of this study was to identify spatial patterns and the relationship between key geographic and environmental factors with the distribution of yellow fever human cases in the Americas.

**Methodology/Principal findings:**

An ecological study was carried out to analyze yellow fever human cases reported to the Pan American Health Organization from 2000 to 2014, aggregated by second administrative level subdivisions (counties). Presence of yellow fever by county was used as the outcome variable and eight geo-environmental factors were used as independent variables. Spatial analysis was performed to identify and examine natural settings per county. Subsequently, a multivariable logistic regression model was built. During the study period, 1,164 cases were reported in eight out of the 13 endemic countries. Nearly 83.8% of these cases were concentrated in three countries: Peru (37.4%), Brazil (28.1%) and Colombia (18.4%); and distributed in 57 states/provinces, specifically in 286 counties (3.4% of total counties). Yellow fever presence was significantly associated with altitude, rain, diversity of non-human primate hosts and temperature. A positive spatial autocorrelation revealed a clustered geographic pattern in 138/286 yellow fever positive counties (48.3%).

**Conclusions/Significance:**

A clustered geographic pattern of yellow fever was identified mostly along the Andes eastern foothills. This risk map could support health policies in endemic countries. Geo-environmental factors associated with presence of yellow fever could help predict and adjust the limits of other risk areas of epidemiological concern.

## Author summary

Yellow fever (YF) is a zoonotic disease caused by yellow fever virus (YFV), which is transmitted to humans through the bite of an infected mosquito. Sylvatic and urban cycles have been present in different periods, but currently most cases result from human exposure to jungle or forested environments. The World Health Organization considers 13 countries endemic for YFV in the Americas. The objective of this study was to identify spatial patterns and the relationship between key geographic and environmental factors with the distribution of YF human cases in the Americas. Cases of YF from 2000 to 2014 aggregated by county and eight geo-environmental factors were studied via spatial and statistical analysis. A total of 1,164 cases were reported in this time period, with the majority of them located in Peru, Brazil and Colombia. Yellow fever presence was associated with rain, altitude, diversity of non-human primate hosts and temperature. A large clustered geographic pattern of YF cases was identified along the Andes eastern foothills. Although YF cases can be seen as rare events, the results of this study demonstrate that YF human cases in the Americas are geographically concentrated and are not happening at random, even within areas known to be at risk. Determining the geo-environmental factors related to YFV is essential to delineate risk areas and to consequently improve resource allocation and prevent human cases.

## Introduction

Yellow fever (YF) is a zoonotic disease caused by an arbovirus of the family *Flaviviridae*, the same family of the dengue and Zika viruses, of which the latter was declared in 2016 a Public Health Emergency of International Concern [1]. YF is caused by yellow fever virus (YFV), which is transmitted to humans through the bite of an infected mosquito, and YF was one of the most feared diseases in the New World before the 20th century [2]. After the discovery of the urban transmission cycle, including the identification of vectors and hosts, and the development of the vaccine in the 1930s, the public fear and epidemiological impact were abridged [3].

The geographic spread of YFV around the world has historic roots in commerce and colonization. Yellow fever was one of the earliest viruses linked to human disease and one of the first for which formal quarantine arrangements were established [4]. From the 15th to 19th centuries, large-scale outbreaks occurred in port cities of North and South America, Africa, and Europe, causing devastating mortality [3,5,6]. The virus was probably introduced to the Americas through ships carrying slaves from West Africa [5], and many colonies in the New World refused the entrance of ships from endemic areas [4].

Sylvatic (jungle) YF is the predominant transmission cycle in the Americas [7]. The cycle involves the circulation of YFV between various species of non-human primates (NHP) and tree-dwelling mosquitoes from the genus *Haemagogus*, the main vector of jungle YF in the Americas, especially *Haemagogus janthinomys* and *Haemagogus spegazzini*, which inhabit the rainforest canopy. *Sabethes chloropterus* mosquitoes are thought to play a secondary role [8]. Humans are usually infected when bitten by sylvatic mosquitoes that previously fed on a viremic monkey [2]. Transmission occurs when non-vaccinated individuals penetrate into areas where the virus is circulating [9] and affects workers whose occupation takes them into the jungle or nearby areas [4, 8]. In rural areas next to forests, the virus causes sporadic outbreaks but, if introduced into urban regions, it can cause large explosive epidemics that are difficult to control [10].

Humans also serve as a viremic amplifying host for inter-human transmission, mainly via the mosquito *Aedes aegypti* (urban yellow fever), a species that breeds in close proximity and often inside human dwellings [2]. Although urban YF seemed to have disappeared from the Americas [8], outbreaks in Paraguay between 2008 and 2009 demonstrated that such risk is still present and will remain as long as the density of its urban vector, the *Aedes aegypti* mosquito, is not under control [8].

Between 1985 and 2009, there were approximately 30,000 cases of YF officially reported to the World Health Organization (WHO); however, this number relies on passive surveillance and thus is significantly underestimated [2]. Currently, YF is endemic in countries in Africa and South America, but over 90% of YF cases occur in Africa, where both the sylvatic (jungle) and urban cycles of the disease still persist [8, 11]. In the course of 2016, YF outbreaks were reported in Angola, the Democratic Republic of the Congo and other African countries, reaching approximately 1,000 confirmed and more than 3,000 suspected cases [10, 12].

In the Region of the Americas, 13 countries are considered endemic for yellow fever by WHO [13, 14]. In North and Central America YF has practically vanished, while in South America it is still found around the Amazon basin, and intermittently on the island of Trinidad [15, 16]. Between 1960 and 1999 there were 5,687 laboratory-confirmed YF cases in the Americas, with the highest numbers reported in Peru (46.7%), Brazil (25.4%) and Bolivia (14.3%) [17]. During this period, 1995 was the year with the highest incidence: 524 cases [17]. French Guiana, Guyana, Panama, Suriname, and Trinidad and Tobago—all endemic countries for YFV—have not reported cases over the past two decades [17]. Large-scale vaccination efforts have been successful in reducing the YF burden of disease for several decades, but vaccination coverage is declining in several countries [10]. In December of 2015, the Pan American Health Organization (PAHO) published an Epidemiological Alert about the risk of YFV and the occurrence of epizootic and human cases in certain areas of the Americas [18].

Since the first half of the 20^th^ century, studies have recognized the importance of having a systemic view, incorporating geographic and environmental factors, to better understand the transmission cycle of disease. Early studies identified natural niches and anthropogenic factors that overlap in time and space, creating biogeographic areas favorable for certain infectious diseases to occur. Some of these factors involve latitude, altitude, orography slope and orientation, land mass size, ecosystem features as rain and temperature, fauna and flora, besides human activity [19, 20].

Many studies focused on the association between mosquito distribution and environmental factors, such as rainfall patterns, temperature and altitude. Kumm et al. described that the *Haemagogus* mosquito is found in areas where the annual rainfall exceeds 2,000 mm [21]. Yellow fever transmission may occur at altitudes up to 2,300 meters in the Americas and possibly higher in Africa [22]. Altitude generates temperature gradients that affect mosquito and virus viability, as well as the distribution of NHPs. Previous research has shown that increases in temperature reduce the extrinsic incubation period of YFV [23, 24]. The ideal temperature for the mosquito incubation period has been recorded to be between 20 and 30 degrees Celsius [25]. During the epidemic of jungle yellow fever in Brazil in 2000, it was observed an increase in temperature and rain during previous months [26].

Recent studies have also confirmed the relevance of geographic factors, such as low latitudes, where infected vectors and hosts overlap with suitable conditions for amplifying the transmission [8, 10]. Low temperature winters in mid-high latitudes, in general, interrupt the disease transmission cycle [3]. Some studies narrow the range of YFV to lower latitudes [8, 27], where vectors and hosts find suitable warmer climates that favor the transmission of the virus [28]. A historical analysis of the global distribution of YF identified that outbreaks occurring from 1900 to 1959 were located between 29°N in the north of Mexico to 29°S in Brazil [4]; in contrast, outbreaks after the 1960s revealed lower latitudes or “intertropical”, from 16°N in Colombia to 28°S in Argentina [4].

In the Americas, the most common NHP involved in the virus sylvatic transmission belong to the genera *Aotus* (owl or night monkeys), *Alouatta* (howler monkeys), *Cebus* (capuchin or white monkeys), *Ateles* (spider monkeys), *Callithrix* (marmosets) and *Saimiri* (squirrel monkeys) [7, 8, 13, 29–31]. Whereas in Africa the majority of simian species have greater resistance to YFV infection and rarely develop the disease, due to long-term adaptation to the virus, in the Americas, neotropical species of monkeys are prone to developing fatal infections [32, 33]. Some are very susceptible and frequently die due to severe disease symptoms, characterized by liver and renal failures and bleeding, especially the *Alouatta* ssp. [8], serving as sentinels for YFV. Others, like the *Aotus* spp., are less exposed to biting of key vectors species due to their nighttime activity [7].

Yellow fever is one of the few diseases for which a certificate of vaccination is required for entry into countries where there is evidence of persistent or periodic disease transmission, regulated under the International Health Regulations [34]. For this reason, mapping and geospatial analysis of risk areas are essential to prevent the spread of the disease and protect countries from YFV importation and individual travelers who might be exposed to the virus [14]. In fact, YF epidemics in the 19th century generated some of the first endeavors in disease mapping [3].

Global mapping efforts exist to identify the boundaries of potential risk areas and vaccination recommendation zones [13, 35]. An international dedicated YF Working Group has met regularly to produce and update a harmonized global risk map with vaccination recommendations. Risk maps are based on environmental conditions, such as elevation, vegetation zones, some serological evidence and available YF case data reported by the countries [36]. Diverse methodological approaches and data have been considered to delimit risk areas, since some countries do not report standardized and geographically detailed information of YF cases among humans and/or infected non-human primates.

Understanding the complexity of ecological interactions in a geographical region is important for prediction, prevention and control measures of vector-borne diseases [37]. The objective of this study is to identify spatial patterns and the relationship between key geographic and environmental factors with the distribution of yellow fever human cases in the Americas.

## Methods

### Study design and data collection

An ecological study design was carried out including the 13 YF-endemic countries of the Americas: Argentina, Bolivia, Brazil, Colombia, Ecuador, French Guiana, Guyana, Panama, Paraguay, Peru, Suriname, Trinidad and Tobago, and Venezuela [13, 14]. Their entire 8,465 second administrative level subdivisions were defined as units of analysis, which in different countries of the region of the Americas are designated as municipalities, provinces, or cantons; but for the purpose of this study were called ‘counties’ [38]. The cartography used was the Second Administrative Level Boundaries (SALB) project, currently under the United Nations Geographic Information Working Group (UNGIWG) [39]. Aggregated data by county was used to analyze the spatial distribution of YF human cases and its relationship with geographic and environmental (hereafter called geo-environmental) factors.

A geocoded database by county was created using different sources of information and variables were shaped/geo-processed from original digital cartography sources and country reports. The dependent variable was the presence of YF human cases by county between 2000 and 2014. Yellow fever human cases are officially reported to the Pan American Health Organization (PAHO) from the Ministries of Health of the endemic countries. Confirmed YF cases reported during the study’s 15-year time period were included in the analysis and geocoded (aggregated by county) in order to standardize the information.

Eight geo-environmental factors were included in the analysis as independent variables: altitude and latitude (essentially geographic); major habitat type, temperature and rain (environmental); hosts (eight genera of non-human primates); proxies of environmental alterations due to human activity (canopy tree loss/disruption and land use intensiveness/agriculture frontier). These map layers and environmental raster data were obtained from several open-access data sources, geo-processed and integrated to each county in the database as variables. For the purpose of this study, all independent variables were considered geo-environmental factors as their spatial distribution and extent were calculated and quantified. The data source used for each variable and how they were measured in the study are described in the S1 File. A hydrography background layer was used as reference when constructing the maps.

### Definitions

The variables in this study were defined as follows:

#### Yellow fever cases

Based on PAHO’s recommended case definition, a probable YF case has one of the following: presence of YFV IgM antibodies in the absence of YFV immunization, within 30 days before onset of illness; positive postmortem liver histopathology; or epidemiological link to a confirmed case or outbreak. All confirmed cases, first defined as probable, need to meet one of the additional criteria: detection of YFV-specific immunoglobulin M (IgM); detection of a fourfold increase in YFV antibody titers between acute and convalescent serum samples; or detection of YFV-specific neutralizing antibodies. A confirmed case can also meet one of the following criteria: detection of YFV genome via polymerase chain reaction (PCR); detection of YFV antigen via immunohistochemical assay; or isolation of YFV. All confirmed cases need to have absence of YFV immunization within 14 days before onset of illness [14]. The YF cases used in this study were confirmed by the Ministries of Health of each country and reported to PAHO at the end of the calendar year, specifying the residence and the probable place of infection.

#### Yellow fever positive counties

Confirmed human cases of YF were geocoded by county based on the probable place of infection. Areas where one or more YF cases were reported during the study period were considered ‘yellow fever-positive counties’.

#### Latitude

Angular distance from the earth’s equator to the county’s calculated centroid was processed by authors. Measured in decimal degrees (°) continuous scale (S1 File).

#### Altitude

Measured as the lowest point within a county in meters above mean sea level (masl) using the global digital elevation model (DEM) from the U.S. Geological Survey and geo-processed by PAHO. Altitude was analyzed as a continuous variable and categorized in four classes using the Jenks natural breaks classification method (S1 File) [40, 41].

#### Major habitat type (MHT)

For the purpose of this study, in the statistical analysis MHT from the World Wildlife Fund ecosystems database was categorized into tropical and non-tropical The tropical classification includes: tropical and subtropical moist broadleaf forests; tropical and subtropical grasslands, savannas and shrublands; tropical and subtropical dry broadleaf forests, and mangroves. Non-tropical classification includes: temperate coniferous forests; savannas and shrublands; temperate broadleaf and mixed forests; montane and temperate grasslands; Mediterranean scrub; deserts and xeric shrublands (S1 File) [42].

#### Temperature

Measured in degree Celsius (°C), BIO1 or Annual Mean Temperature from the WorldClim database during a period of 30 years. The mean annual temperature was geo-processed by each county, analyzed first in a continuous scale and afterwards using four natural break classes (S1 File) [43, 44]

#### Rain

Measured in millimeters (mm) BIO12 or Annual Precipitation from the WorldClim database during a period of 30 years. The total annual precipitation was geo-processed for each county and initially analyzed in a continuous scale and then using four natural break classes (S1 File) [43, 44].

#### Land use intensiveness or frontier (proxy of agriculture frontier)

In order to study the effect of human activity on the environment, we analyzed land use categorizing its use intensity as a proxy of agricultural frontier over natural areas. This variable was constructed by combining natural land use and use-intensity from 40 categories of the Land Use Systems of the World for Latin American & the Caribbean (S1 File) [45, 46]. “Frontier” resulted from selecting high to moderate use intensiveness of from the original natural areas.

#### Tree canopy loss

Measured in percent (%) of municipal area with canopy tree loss or disruption over 30% between 2000 and 2012 from the Global Forest Change maps (S1 File) [47, 48].

#### Non-human primates (NHP)

A mammal from the primate order that is not human, such as apes and monkeys. The number of different genera of NHP hosts (*Alouatta*, *Aotus*, *Callithrix*, *Saguinus*, *Ateles*, *Cebus*, *Saimiri*, *Lagothrix*) by county were calculated based on the terrestrial mammal digital cartography and the databased provided by the International Unit for Conservation of Nature. (S1 File) [49]. No census of NHP by county was found in open-access databases.

### Geographical features and environmental digital cartography

A set of cartographic digital databases and attributes were assembled and shaped using different sources. Digital cartography of counties’ boundaries was previously compiled by PAHO from various countries’ national cartographic agencies (e.g. census offices, military, geographic or national statistics agencies), they were standardized, updated and geocoded following the original guidelines of the SALB project in the context of the cartographic activities of the WHO, currently under the United Nations Geographic Information Working Group (UNGIWG) (S1 File) [39]. Further sets of environmental digital cartography were obtained from diverse public sources depending on the nature of the variable, geo-processed and incorporated to each county subdivision (S1 File).

### Data geoprocessing

The digital cartographic database by county was prepared to aggregate all YF human cases during the study period and the geo-environmental variables of the study. Geocoding of individual YF cases by county and other spatial processing techniques (listed below) were used to assign the geo-environmental statistical information to the county digital database using ArcGIS 10.4.

- Calculating geometry was used to measure latitude of the county’s polygon centroid.
- Zonal statistics (min, mean, max, standard deviation, range) were calculated to measure counties’ lowest altitude, mean temperature and mean total annual rain.
- Zonal statistics\majority technique was applied for measuring the land use/frontier class that occupies the largest surface of the county.
- Map overlapping technique/geoprocessing intersect was used to delineate and calculate the extent (surface in Sq. km and %) of environmental features as MHT Ecosystems and NHP digital databases. Presence of Tropical MHT and NHP by county were identified.

Natural breaks thematic mapping was produced to classify the geo-environmental variables and to determine class limits for independent variables. Proximity techniques were used to identify the YF-positive counties first-order contiguous neighbors [50].

### Spatial patterns detection—Cluster analysis

The ArcGIS 10.4 spatial autocorrelation methods Global Moran’s I and Anselin Local Moran’s I were applied to detect and locate clusters of YF-positive counties. For this purpose, the inverse distance (IDW) approach was used [50]. As most of digital cartographic data sources were available in the latitude-longitude system, (WGS_1984 EPSG 4326), a customized cartographic projection, Azimuthal Equidistant (WKID: 54032) was applied adjusting central meridian to -80 degrees longitude and 10 degrees for origin latitude, to reduce distances distortion at continental level [51, 52].

### Statistical data analysis of geo-environmental factors associated to YF presence

Once the spatial calculations were integrated into the county attributes database, data were analyzed with R statistical software (version 3.0.0). The dependent variable was dichotomized according to the presence of YF reported during the study period: coded as 1 if the county had reported at least one case during the last 15 years or 0 if no cases were reported during this time. Geo-environmental related factors (described previously) were included in the analysis as independent variables.

Cross tabulation and descriptive statistics such as median, interquartile range and frequency were performed for all independent variables. To describe and analyze the independence between positive and negative counties, Mann-Whiney *U* test was used to measure the difference between presence and absence of YF. Independent variables were first screened based on the response variable; in the case of variables with large amounts of missing data (>10%) and limited variability (coefficient of variation <20%), they were not included in the multivariable model. The variables were then entered individually into a univariate logistic regression model and preselected if p-value ≤ 0.15. Subsequently, variance inflation factor (VIF) was estimated to verify the relationship between all preselected independent variables (check for potential collinearity), in which coefficient >10 was considered high. For this study none VIFs were higher than 10. Interactions between biologically plausible variables were examined (rain vs. temperature; MHT vs. canopy tree disruption or loss and MHT vs. precipitation), if found significant (p <0.05), interaction terms were kept for further analysis.

Eight independent variables were included in the initial multivariable model: latitude (continuous), altitude (categorical-natural breaks), tropical MHT (categorical-dichotomous), temperature (categorical-natural breaks), annual mean rain (categorical-natural breaks), number of genera of NHP hosts (continuous), land use intensiveness/frontier (categorical-dichotomous), and canopy tree loss (continuous). Multivariable models were built in a manual stepwise fashion starting with the forward method; where each remaining variable was added to the best previous model, selected by the Akaike Information Criterion (AIC); in the case the variable remained numerically the same, the Bayesian Information Criterion (BIC) was used. Lastly, a backward elimination step was performed, resulting in a final model in which only variables with p <0.05 were kept. Confounding effects were investigated by checking changes in the point estimates of the variables that were kept in the model. Changes in parameter estimates higher than 25% were considered as indicative of confounding and if present it was properly controlled by keeping the variables in the model throughout the selection process. The goodness-of-fit of the final model was tested using Hosmer-Lemeshow, p>0.05 [53].

In addition, a mixed effect model approach was conducted in order to explore the different countries (random effect), since we expected that cases of YF may lack independence among countries. We calculated the intra class correlation (ICC) of country as a random effect and the ICC was 0.041, which means that ~4% of the variance can be attributed to the countries (S2 File). Based on this result, we did not consider using country as random effect for our candidate models.

## Results

### Exploratory and descriptive analysis

A total of 1,164 confirmed YF human cases were reported in the Americas between 2000 and 2014 with an average of 78 cases per year (Table 1). Out of the 13 endemic countries, five did not report YF human cases during the 15-year study period: French Guiana, Guyana, Panama, Suriname, and Trinidad and Tobago. The year 2003 presented the highest number of cases in the time period (234 cases). Nearly 83.8% of the total number of cases in the region was concentrated in three countries: Peru (435 cases, 37.4%), Brazil (327 cases, 28.1%) and Colombia (214 cases, 18.4%).

**Table 1:** Number of yellow fever human cases by country and first administrative level subdivision, Americas, 2000–2014.

State/Province by Country	2000	2001	2002	2003	2004	2005	2006	2007	2008	2009	2010	2011	2012	2013	2014	Total
San Martin	3	12	21	12	16	28	6	7	5	6	10	6	2	5	6	**145**
Junin	1	1	12	2	33	4	19				1	1	1	3	2	**80**
Amazonas			2			31	12		7				1			**53**
Cuzco	1		6	6	1		3	10	1	1	5		1	1		**36**
Loreto		10	6		2	1	7	2	1	1		2	1	2	1	**36**
Madre de Dios			2	4	8		1	1	1		1	2	2	3		**25**
Puno			2	1		1	7	1			1		4	2		**19**
Huanuco	1	2			8			1							2	**14**
Ucayali		3				1						1	2	3	1	**11**
Ayacucho		1	1				4					1	1	2		**10**
Pasco							4	1						1		**6**
**Peru**	**6**	**29**	**52**	**25**	**68**	**66**	**63**	**23**	**15**	**8**	**18**	**13**	**15**	**22**	**12**	**435**
Minas Gerais	2	32	8	56					1	1						**100**
Goias	53							7	17							**77**
Amazonas	1	3	6		3	2	1	2						2		**20**
São Paulo	2								2	28						**32**
Rio Grande do Sul									6	15						**21**
Mato Grosso	7	2		4	1		1	1	2	2						**20**
Pará	1	2	1	1	2			1	3		1			1	1	**14**
Bahia	10															**10**
Mato Grosso do Sul									9		1					**10**
Distrito Federal	2							1	5							**8**
Tocantins	6															**6**
Roraima		1	2			1		1								**5**
Paraná									2							**2**
Acre	1															**1**
Rondônia		1														**1**
**Brazil**	**85**	**41**	**17**	**61**	**6**	**3**	**2**	**13**	**47**	**46**	**2**			**3**	**1**	**327**
Norte de Santander				91	2		1									**94**
Guaviare	2	4	12	1		1			1							**21**
Meta	2	1	1	2	2	1	1	3	2	5						**20**
Cesar				5	13											**18**
Magdalena				3	12											**15**
Caquetá				1		9		1								**11**
Putumayo						8	1									**9**
Vichada	1	1	3		1		1	1								**8**
Casanare			3	2			1	1								**7**
La Guajira				2	4											**6**
Amazonas		1	1													**2**
Santander				1		1										**2**
Guainía		1														**1**
**Colombia**	**5**	**8**	**20**	**108**	**34**	**20**	**5**	**6**	**3**	**5**						**214**
Zulia			3	21												**24**
Táchira				11												**11**
Portuguesa				2		7										**9**
Merida					2	3										**5**
Monagas					3											**3**
Apure						1										**1**
Bolivar						1										**1**
**Venezuela**			**3**	**34**	**5**	**12**										**54**
Cochabamba	1		4	2	3	13	10	1				2	2			**38**
Santa Cruz	5		6	1	8	1	3									**24**
La Paz	1	1	2	1	2	2	3	5	1		3				1	**22**
Beni	1	2	2	2									1			**8**
Chuquisaca			1													**1**
Pando		1														**1**
**Bolivia**	**8**	**4**	**15**	**6**	**13**	**16**	**16**	**6**	**1**		**3**	**2**	**3**		**1**	**94**
San Pedro								1	14							**15**
Central									9							**9**
Caaguazú									4							**4**
**Paraguay**								**1**	**27**							**28**
Misiones									8	1						**9**
**Argentina**									**8**	**1**						**9**
Napo	2												1			**3**
**Ecuador**	**2**												**1**			**3**
**Total**	**106**	**82**	**107**	**234**	**126**	**117**	**86**	**49**	**101**	**60**	**23**	**15**	**19**	**25**	**14**	**1,164**

A total of 57 out of 732 first administrative subdivisions (i.e. states/provinces/departments) of the countries included in the study reported YF cases. The highest numbers were found in San Martin, Peru with a total of 145 cases during the study period, (12.5% and had cases every year of the studied period), Minas Gerais, Brazil with 100 cases (8.6%), Norte de Santander, Colombia with 94 cases (8.1%), Junín, Peru with 80 cases (6.9%), and Goias, Brazil with 77 cases (6.6%).

**Fig 1:**
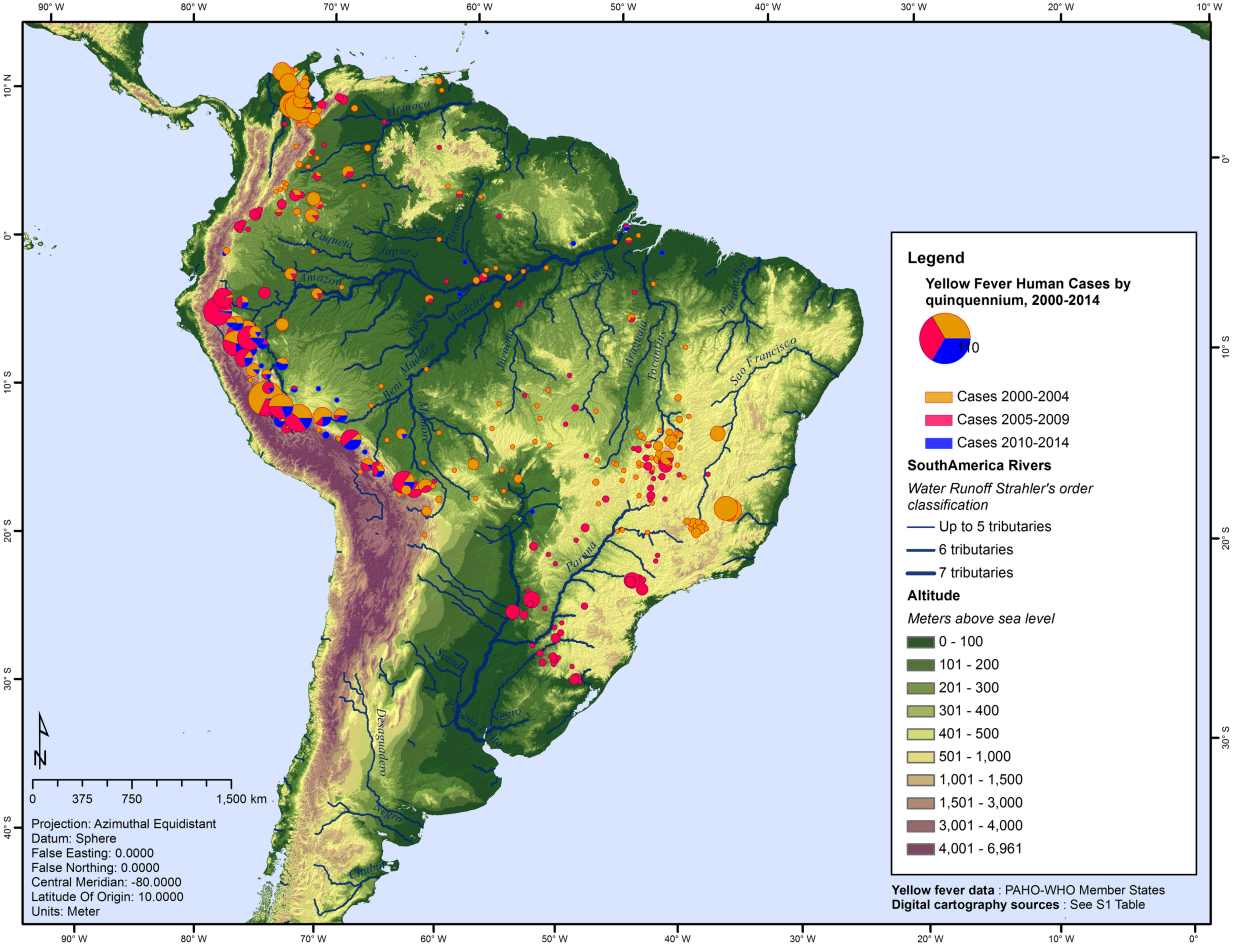
Geographic distribution of counties with yellow fever cases by quinquennial, Americas, 2000–2014.

Yellow fever was present in 286 counties during the study period, which represent merely 3.4% out of the total 8,465 counties studied. A large group of YF-positive counties was found along all the Andes eastern foothills during the 15 years of the study period (Fig 1), at the upper basin of the Amazon River and its main tributaries (Marañon, Ucayali, and Madre de Dios). Another noteworthy group of cases was identified in the north of South America between the Magdalena River and the Maracaibo Lake recorded mostly during the 2000–2004 quinquennial. The Orinoco basin displays scattered YF-positive counties. In the Brazilian highlands, at the very upper basins of the rivers Araguaia, Tocantins, San Francisco and Doce, groups of YF-positive counties were identified until 2009; fewer counties reported YF cases in the 2010–2014 quinquennial. Between 2005 and 2009, there were reported cases of YF in counties located along the Paraná-Paraguay, the Uruguay and the Jacui and Cai rivers, all flowing towards the Southern Atlantic Ocean. Along the main course of the Amazon River there were scarcer positive counties.

Yellow fever-positive counties were located between latitudes of 11.3 degrees north and 29.7 degrees south, registering a median latitude of 12.5 degrees south (Fig 1). Counties without YF had a median latitude of 14.02 degrees south. There was a significant statistical difference between counties with and without YF (Mann-Whitney *U* = 136, *p* <0.001) and YF-positive counties had a median latitude ~2 degrees closer to the Equator.

**Fig 2:**
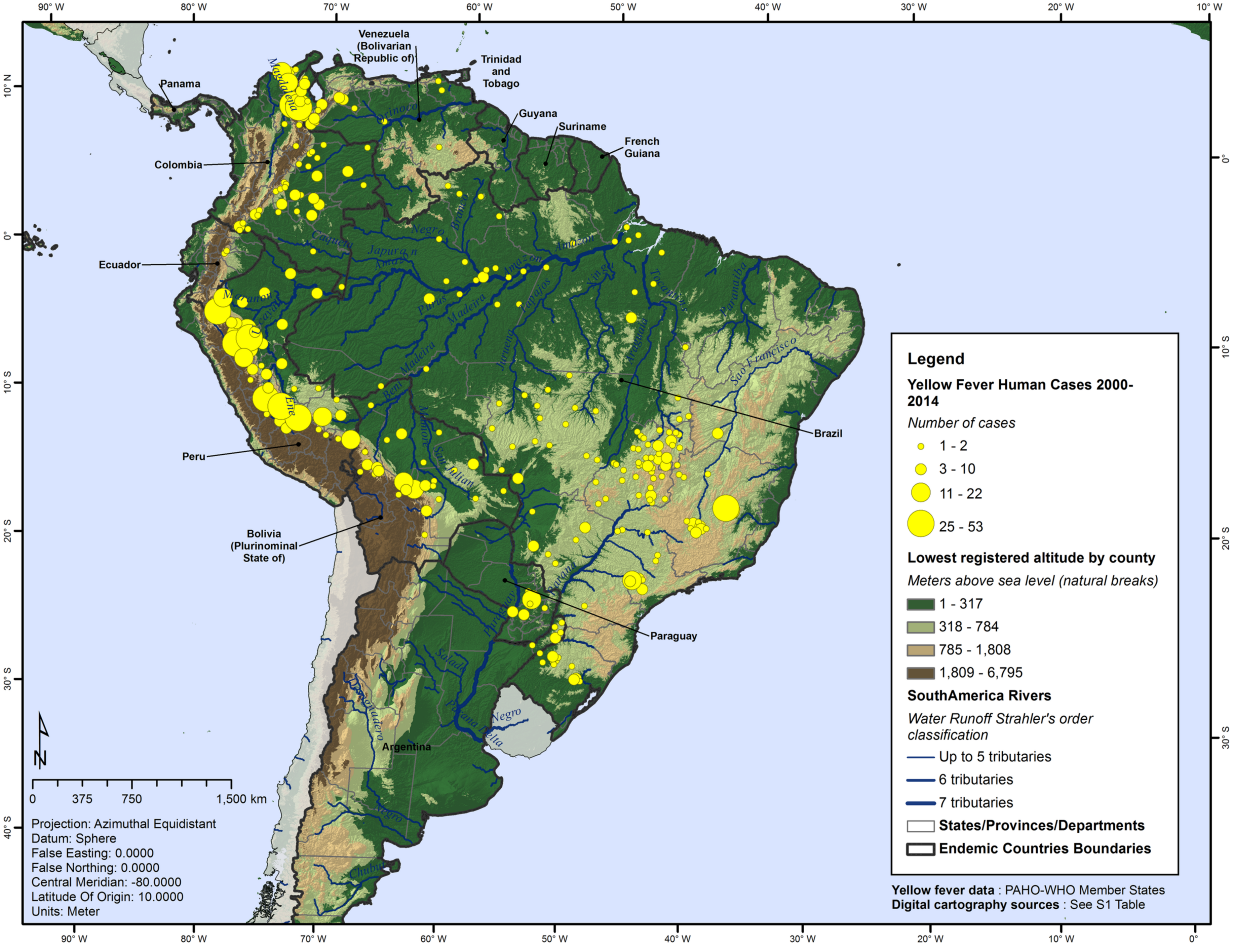
Lowest registered altitude by county and geographic distribution of yellow fever, Americas, 2000–2014.

Altitude in the study area fluctuates from sea level, as the rivers outlets of the Amazon and its large tributaries, to the elevations of the Andes, including the Aconcagua Mountain as the highest point with 6,961 meters above sea level (masl) (Fig 2). YF counties registered altitudes between 1 and 3,259 masl, with a median of 237 masl and an interquartile range from 92.3 to 459.5 masl. When compared with counties without YF cases, which median is 277 masl, no statistically significant difference (40 meters) was found between groups (Mann-Whitney *U* test = 12, *p* = 0.08).

**Fig 3:**
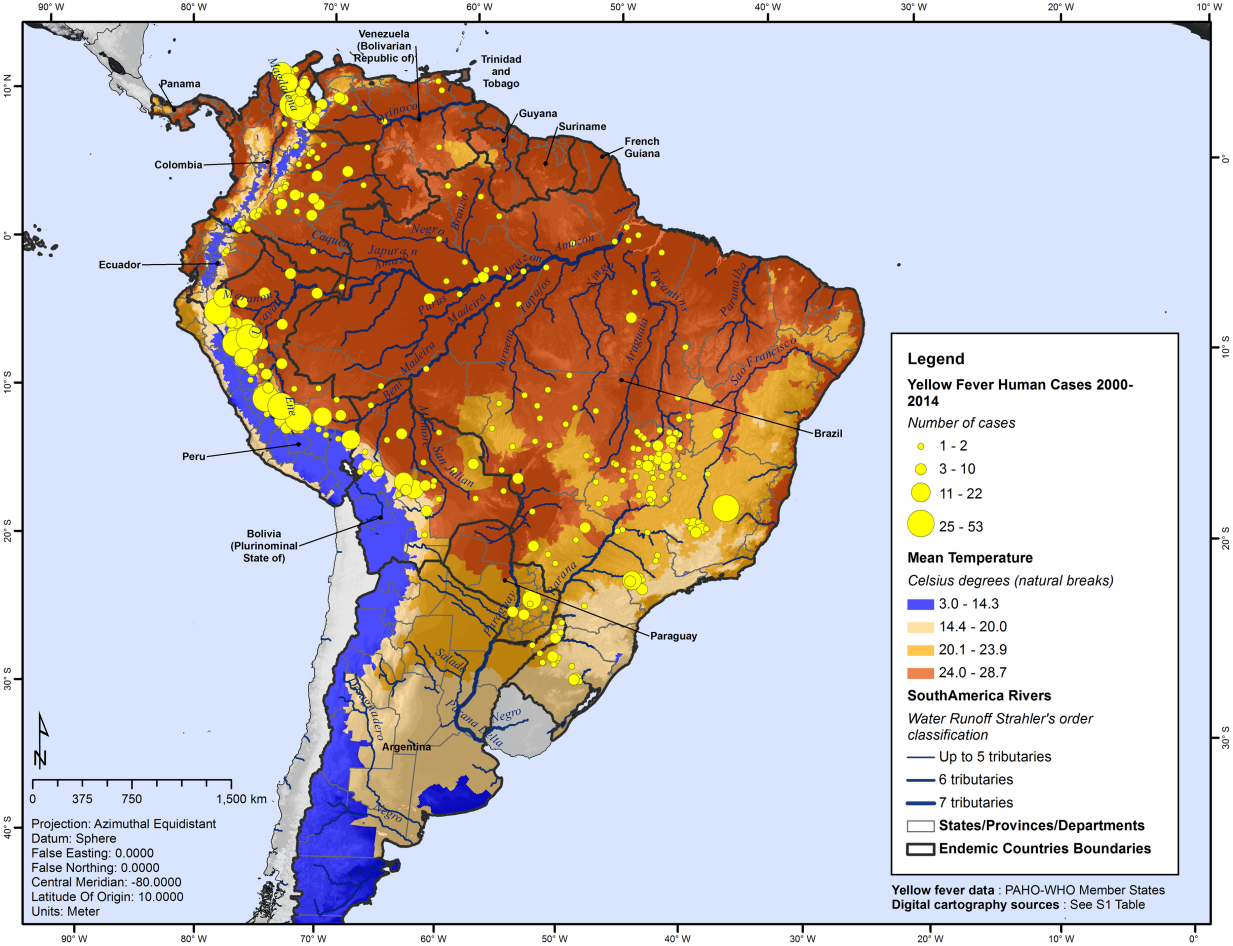
Mean temperature by county and geographic distribution of yellow fever, Americas, 2000–2014.

In the case of temperature conditions, the whole study area had a median annual mean temperature of 22.2°C (Fig 3). Yellow fever positive counties registered a median temperature of 24.1°C, ranging from 5.9°C to 28.5°C. A significant difference between groups was found when comparing counties with and without YF cases, median temperature = 22.1°C (Mann-Whitney *U* test = 87, *p* <0.001). This finding suggests that annual median temperature in YF-positive counties is ~2 degrees above the temperature in counties without YF cases.

**Fig 4:**
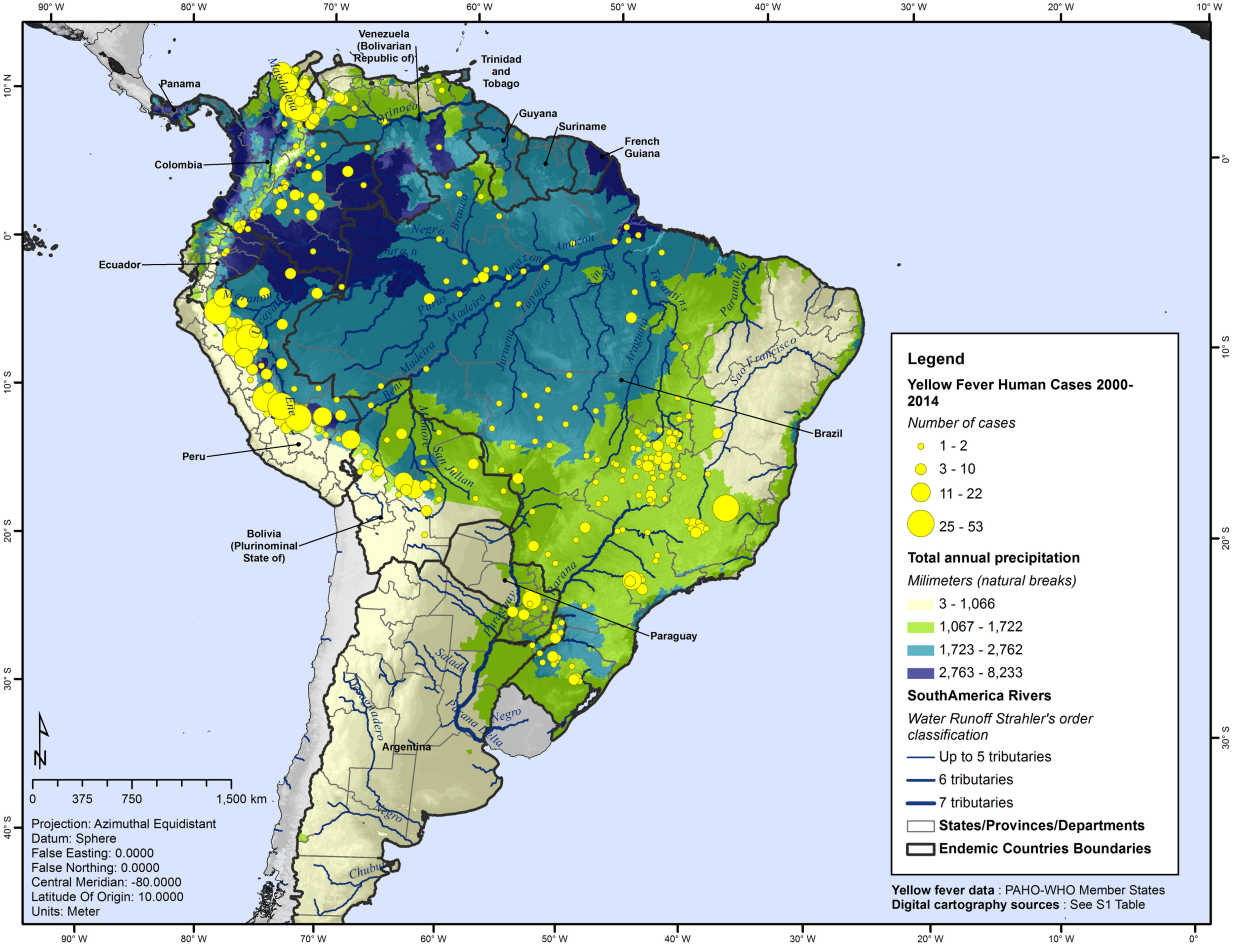
Total annual rainfall by county and geographic distribution of yellow fever, Americas, 2000–2014.

The median county rainfall in the study area was of 1,384 mm (Fig 4). In YF-positive counties a median rainfall of 1,681 mm was observed, ranging from 566 mm to 3,809 mm a year. When compared with counties without YF cases, a significant difference was observed between groups (Mann-Whitney *U* test = 66, *p* <0.001). The median annual rainfall in YF counties was 308 mm more abundant than in counties without YF cases.

**Fig 5:**
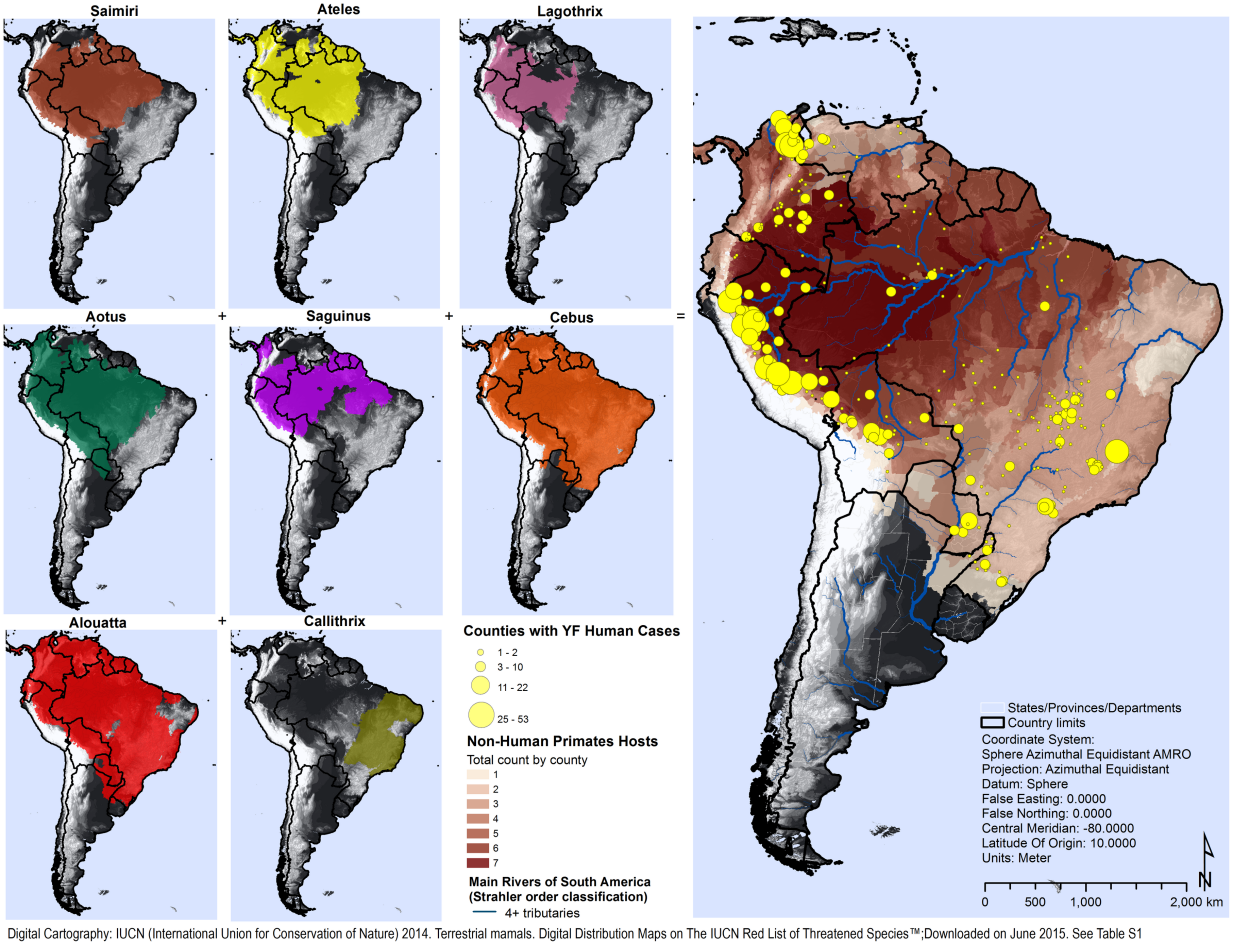
Number of NHP genera of potential YFV hosts by county and geographic distribution of YF, Americas, 2000–2014.

There is a large diversity of primates in the study area. Based on the literature review, eight NHP genera were identified as possible YFV hosts in the 13 endemic countries of the Americas: *Alouatta*, *Aotus*, *Ateles*, *Callithrix*, *Cebus*, *Lagothrix*, *Saguinus* and *Saimiri*. The geographic overlap of the different genera is most predominant in the middle and upper Amazon River basin, in its tributaries Madeira River and Negro River in the central Amazon region in Brazil and upstream the Ucayali and Maranon rivers in Peru (Fig 5).

The median count of different genera of NHP hosts by county was three for the entire study area (range: 0–7) and four for YF-positive counties. There were no counties in the study with eight different genera of NHP hosts. Compared to counties with no YF cases (median of two NHP genera), we found a significant difference between groups (Mann-Whitney *U* test = 59, *p* <0.001). Yellow fever counties registered a median of two additional genera of NHP hosts than counties with no YF cases in the study period.

With geographic proximity techniques we identified 791 contiguous neighboring counties with no reported YF human cases, among which we found similarities—using Mann-Whitney U test- with YF counties in terms of tropical habitat and land use intensity (frontier), whereas contrast in latitude, altitude, temperature & rain patterns, as well as in the number of genera of NHP hosts (S3 File).

### Univariate analysis

Table 2 presents the results of the univariate analysis (*p* < 0.15) using logistic regression to measure possible relationship between yellow fever positives counties and eight geo-environmental factors.

**Table 2:** Univariate analysis of possible geo-environmental factors contributing to the presence of yellow fever human cases, Americas, 2000–2014.

Variable	No. (%)/Median (IQR)	p-value	Odds Ratio (CI _95%_)
**Altitude (masl**^a^**)**			
0–317	4,716 (55.7%)	0.01	**5.88** (1.45–23.80)
318–784	2,620 (30.9%)	0.01	**5.33** (1.30–21.74)
785–1,808	834 (9.8%)	0.45	1.77 (0.38–8.16)
≥ 1,809	295 (3.5%)	Ref	Ref
**Land use intensiveness/ frontier**			
Low intensity use	6,101 (72.1%)	Ref	Ref
High-moderate intensity Use	2,364 (27.9%)	0.003	**1.56** (1.22–2.00)
**Latitude (°)**	-13.850 (-22.800- -3.584)	0.02	**0.96** (0.95–0.97)
**Major habitat type**			
No tropical	1,807 (21.3%)	Ref	Ref
Tropical	6,657 (78.6%)	<0.001	**7.77** (4.12–14.63)
**Rain (mm)**			
3–1,066	2,215 (26.2%)	Ref	Ref
1,067–1,722	4,256 (50.3%)	<0.001	**7.44** (3.91–14.17)
1,723–2,762	1,697 (20.1%)	<0.001	**15.73** (8.21–30.12)
2,763–8,233	297 (3.5%)	<0.001	**19.38** (9.17–40.96)
**Temperature (°C)**			
3.0–14.3	536 (6.3%)	Ref	Ref
14.4–20.0	2,147 (25.4%)	0.70	1.15 (0.53–2.49)
20.1–23.9	2,943 (34.8%)	0.03	**2.22** (1.07–4.60)
24.0–28.7	2,839 (33.5%)	<0.001	**3.55** (1.73–7.28)
**Number of genera of NHP hosts**	3 (0–7)	<0.001	**1.80** (1.68–1.94)
**Tree canopy loss**	76.34 (41.76–95.88)	0.07	0.98 (0.97–1.08)

^a^ Meters above mean sea level

#### Rainfall

Compared to the other geo-environmental variables, rain presented the highest odds ratio (OR) and all classes were statistically significant. The odds of being a YF-positive county increases with the precipitation level, compared with drier areas. The OR range from 7.44 (CI_95%_ = 3.91–14.17) to 19.38 (CI_95%_ = 9.17–40.96) in the highest rain quartile (2,763–8,233 mm).

#### Major habitat type

Approximately 79% of the YF-positive counties in the study have some portion of tropical habitat within their boundaries. The odds of reporting YF was 7.77 higher when compared with non-tropical (*p* <0.001).

#### Altitude

Using as a reference altitude above or equal to 1,809 masl, counties located at an altitude between 0–317 masl were 5.88 times more likely to be YF-positive (*p* = 0.01). For those counties located at an altitude from 318 to 784 masl, the odds was 5.33 (*p* = 0.01). No significant difference was found when comparing counties between 785–1,808 masl with higher altitudes.

#### Temperature

Higher temperatures increased the odds of being a YF county. Counties where the mean annual temperature ranges between 20.1–23.9°C and 24.0–28.7°C, shown an odds ratio of 2.22 and 3.55 times higher of being a YF-positive county compared to the reference group (counties with mean annual temperature between 3.0–14.3°C).

#### Non-human primates

The univariate analysis show that for every one additional genus of NHP host present in the area, there was an increase in the odds of being a YF-positive county Table 3 presents a more detailed analysis for each NHP host present in the study region. Two genera of NHP hosts are abundant in the study area: *Cebus* spp. is present in 82% of the counties (6,943 counties) and *Alouatta* spp. in nearly 79% (6,642 counties). Seven out of eight genera of NHP hosts studied presented a positive statistically significant association, from an OR of 3.35 (*p* <0.001) for *Cebus* spp. to 7.06 (*p* <0.001) for *Saimiri* spp. *Cebus* and *Alouatta’s* presence exhibited similar odds of 3.35 and 3.56 respectively (*p* <0.001). Presence of *Callithrix* spp. was negatively associated with the occurrence of YF.

**Table 3:** Univariate analysis of non-human primate hosts studied in the region, Americas, 2000–2014.

Non-human primates	No.(%)/Median (IQR)	p-value	Odds Ratio (CI _95%_)
***Alouatta* spp.**			
Absence	1,823 (21.5%)	Ref	Ref
Presence	6,642 (78.5%)	<0.001	**3.56** (2.27–5.77)
***Aotus* spp.**			
Absence	6,269 (74.1%)	Ref	Ref
Presence	2,196 (25.9%)	<0.001	**4.73** (3.71–6.02)
***Ateles* spp.**			
Absence	7,217 (85.3%)	Ref	Ref
Presence	1,248 (14.7%)	<0.001	**5.42** (4.26–6.90)
***Callithrix* spp.**			
Absence	5,272 (62.3%)	Ref	Ref
Presence	3,193 (37.7%)	<0.001	**0.56** (0.43–0.74)
***Cebus* spp.**			
Absence	1,522 (18.0%)	Ref	Ref
Presence	6,943 (82.0%)	<0.001	**3.35** (2.07–5.42)
***Lagothrix* spp.**			
Absence	7,784 (92%)	Ref	Ref
Presence	681 (8%)	<0.001	**4.71** (3.58–6.19)
***Saimiri* spp.**			
Absence	7,424 (87.7%)	Ref	Ref
Presence	1,041 (12.3%)	<0.001	**7.06** (5.54–9.00)
***Saguinus* spp.**			
Absence	7,506 (88.7%)	Ref	Ref
Presence	959 (11.3%)	<0.001	**3.79** (2.92–4.92)

#### Land use intensiveness/frontier

We found that approximately 28% of the counties under study have a high to moderate land use intensity of natural environments, which increases the odds of having a YF county by 56% in comparison with natural areas where land use intensity is low or no use is reported.

#### Latitude

Based on the univariate analysis, higher latitude was negatively associated with YF. For every one degree increase, North or Southbound, there is a 4% decrease in the odds of being a YF-positive county (OR = 0.96; *p* <0.001).

#### Tree canopy loss

No significant effect was found in tree canopy loss per county.

### Multivariable analysis

The final logistic regression model identified four significant geo-environmental factors associated with the presence of yellow fever human cases (*p* ≤ 0.05): rain, altitude, number of genera of NHP hosts and temperature (Table 4).

**Table 4:** Final multivariable analysis of geo-environmental factors associated with the presence of yellow fever human cases, Americas, 2000–2014.

Variables	p-value	Odds Ratio (CI _95%_)
**Altitude (masl**^a^**)**		
0–317	0.05	5.13 (0.96–27.29)
318–784	0.02	**6.76** (1.29–35.23)
785–1,808	0.67	1.41 (0.27–7.17)
≥ 1,809	Ref	Ref
**Number of NHP genera**	<0.001	**1.81** (1.63–2.02)
**Rain (mm)**		
3.0–1,066	Ref	Ref
1,067–1,722	<0.001	**4.23** (2.17–8.23)
1,723–2,762	<0.001	**4.22** (2.05–8.71)
2,763–8,233	0.05	**2.34** (1.00–5.53)
**Temperature (**°C)		
3.0–14.3	Ref	Ref
14.4–20.0	0.005	**4.12** (1.51–11.27)
20.1–23.9	0.90	0.96 (0.59–1.58)
24.0–28.7	0.15	1.30 (0.90–1.87)

^a^ Meters above mean sea level

Altitudes between 318–784 masl were significantly associated with YF presence (OR = 6.76) when compared to altitudes greater or equal to 1,809 masl. Altitudes from 0 to 317 masl were not considered since the CI included the null.

Rainfall was associated with higher odds of YF, especially in counties with 1,067–1,722 mm and 1,723–2,762 mm (OR = 4.23 and 4.22, respectively); amounts of rain higher than 2,763 mm had a marginally significant association with a lower odds ratio of 2.34.

Counties with moderate annual temperatures between 14.4–20.0°C had an OR of 4.12 for a yellow fever positive county compared to the reference group (counties with mean annual temperature between 3.0–14°C). Temperatures higher than 20.1°C were not statistically significant.

The number of different genera of NPH hosts by county was significantly associated with YF presence (OR = 1.81).

The final model had a good fit to the data using Hosmer-Lemeshow test [53].

### Spatial patterns—Cluster analysis

Among the YF-positive counties a spatial autocorrelation was observed exposing clustered areas with comparable number of YF cases. Moran’s value (I = 0.02; *p* <0.001; z-score = 19.89). The Global Moran’s I detected that 2% of the total counties in the study area showed significant clustering.

**Fig 6:**
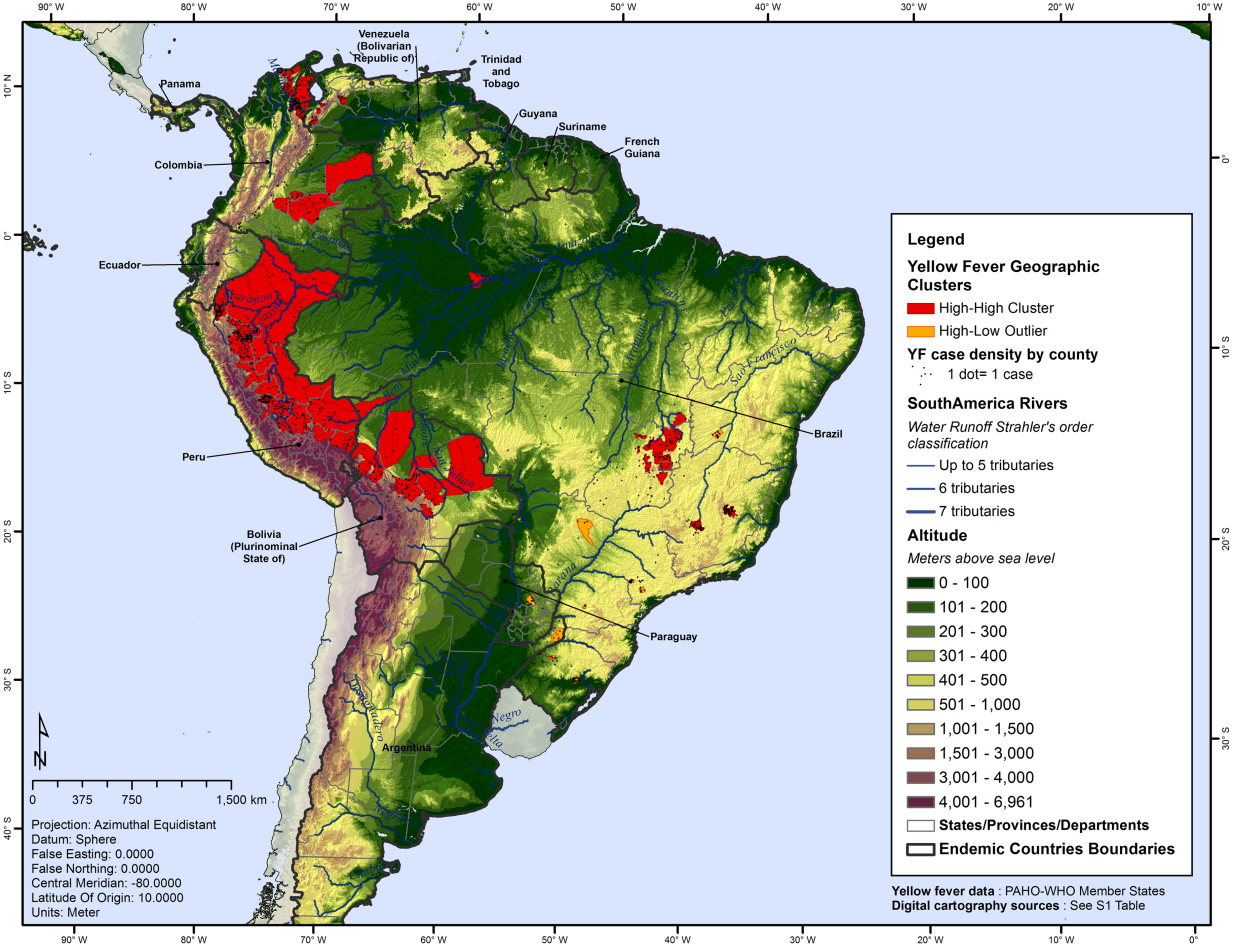
Yellow fever geographic clusters by county, Americas, 2000–2014.

Anselin Local Moran’s I identified and located a total of 138 statistically significant clustered counties with 962 YF human cases (82.6%). They were characterized as follows: 127 YF counties were classified as high-high clusters (counties with high number of cases, where neighboring counties also have high YF values); and 11 as high-low outliers (counties with high number of cases among low YF value neighbors) (Fig 6).

The remaining 148 YF-positive counties were not significantly clustered and were distributed throughout Brazil, Colombia and Paraguay, accounting for 17.4% of total number of YF cases in the study period (202 cases).

Most high-high clusters were located in the Peruvian Andes eastern foothills and alongside intermountain river valleys, large Amazon tributaries like the Marañon and Ucayali. These clustered counties were geographically concentrated in 11 departments of Peru: Loreto, Amazonas, San Martin, Ucayali, Huánuco, Pasco, Junín, Madre de Dios, Cusco, Ayacucho and Puno. As a proximate extension of the Peruvian high-high cluster, Bolivia showed contiguous YF clustered areas in La Paz, Beni, Cochabamba and Santa Cruz. A bi-national high-high cluster was found in the border between Colombia (Norte de Santander, La Guajira and Cesar) and Venezuela (Zulia). Another high-high cluster was found in the South of Colombia, including counties in the departments of Guaviare, Guainía, Vichada, and south of Meta. Brazil had a contiguous high-high cluster including the states of Minas Gerais, another in Goias, the Federal District and south of Tocantins, and an isolated cluster in the state of Amazonas. Relatively smaller clusters were detected in São Paulo, Bahia, Paraná and Rio Grande do Sul. Mato Grosso do Sul in Brazil, Paraguay and Argentina had high-low clusters. No statistically significant low-high or low-low clusters were found in the whole area. The state of Para was not identified within a cluster, but presented YF during ten of the 15 years included in the study.

## Discussion

This study identified geographic patterns and key geo-environmental factors associated with the distribution of YF human cases in the Americas: altitude (between 318 and 784 masl), annual rainfall (between 1,067 and 2,762 mm), temperature (between 14.4°C and 20.0°C) and number of genera of NHP hosts. There is also sufficient evidence to conclude that the presence of YF in South America is not a series of isolated events and is not happening at random across the study area, as spatial clustered patterns were discovered and characterized.

Previous studies have acknowledged that altitude has a leading role associated with YF presence, because it generates temperature gradients that affect mosquito and virus viability [28], as well as NHP location. A previous study in Colombia about the distribution of the *Haemagogus* mosquito in the sylvatic cycle found that the vector is abundant at altitudes below 2,000 meters above sea level [54]. In Brazil, however, where most of the country has an elevation below 1,000 meters, the effect of altitude is not so pronounced [21]. In this study we found that counties between 318 and 784 masl had six times higher risk of YF compared to counties at higher altitudes.

Climatic elements, such as rainfall and temperature, are key elements that define YF geographic patterns. Intertropical/Equatorial climates are characterized by regimes of warm temperatures and abundant rainfall patterns [55]. In this study, counties with annual rainfall between 1,067 and 2,762 mm had four times higher odds of YF. The Andes eastern foothills receive constant moisture that trade winds bring to the inward continental mass, enhancing conditions for orographic precipitation, source of water in the large Amazon basin; even in driest months, these areas registered large amounts of rainfall compared with the remaining YF endemic regions [56]. Peru, located in the Andes eastern foothills, has areas that reported cases during the whole year (S4 File).

Away from the Equator, seasonality can play a more important role. Studies in Trinidad showed that density of *Haemagogus janthinomys* mosquitoes were about six times greater during the wet season (May-November) than in the dry season [57]. In addition, *Haemagosgus janthinomys’* larval abundance has been recorded to peak in the rainy season [58]. A research developed in the tropical area of the Caxiuanã National Forest, state of Pará in Brazil (two degrees south of the Equator), which studied *Haemagogus* and *Sabethes* mosquitos and the role of microclimates, found that there is a larger number of vector species during the wettest months, but the difference between seasons was not statistically significant. In the same study, the number of *Hg*. *janthinomys* was positively correlated with variations in temperature and relative humidity [59].

Even though the effect of seasonality on YF was not the objective of this study, it could be important to have a closer examination of the relationship between latitude and seasonality, since the majority of YF-positive counties are located near the Equator. Additional studies are suggested to better understand the seasonal variation according to the latitude in the vast territory of the South American Region (S5 File), as well as to investigate the effect of time patterns, climate change and the El Niño Southern Oscillation on YF and other arbovirus.

The effect of temperature on the expected life span of mosquitoes is also an important factor. Studies have shown that high temperature lead to higher mosquito abundance and consequently an increase in viral circulation. The lowest temperature that YF infectiousness has been observed to develop in a mosquito is approximately 16.5°C [60]. Conversely, temperatures greater than 35°C negatively affect *Aedes aegypti* activity and survival [23, 61]. Temperature has long been known to influence the extrinsic incubation period of YFV in *Ae*. *aegypti* and statistical models have been developed to estimate it [23]. Extreme temperatures negatively affect YFV. Our study showed that the county’s temperature favorable for YF presence ranged from 14.4°C to 20.0°C. Future studies should be developed to measure more precisely the temperature threshold for YF human cases.

The ecology of YFV is complex. Mosquitoes and vertebrate NHP hosts coexist and dwell in the same habitat during the same season. Species of *Haemagogus* and *Sabethes* mosquitoes have been collected in forest locations where sylvatic YF occurs among monkeys [25]. According to the literature, all neotropical NHP are susceptible and considered YFV reservoirs in wild regions [62]. Eradication of YF in the tropical forest environments is almost impossible due to the widespread wildlife reservoir [10].

In the final model of this study, for every one additional genus of NHP host present in the area, the odds of YF occurrence doubled, suggesting that primate diversity can be associated with environmental factors that favor the presence of YF human cases. Future studies are needed to understand the behavior and the role in the transmission of different genera of NHP hosts present in YF areas. Howler monkeys (*Alouatta* spp.*)*, which are extremely susceptible to YFV and develop fatal disease[63], and white monkeys (*Cebus* spp.) occupy most of the area studied in this paper. Spider monkeys (*Saimiri* spp.), which can carry the virus to distant places and also the night monkey (*Aotus trivirgatus*), who are less exposed to YFV due to their nighttime activity, are less abundant in South America. The majority of recent publications about NHP and YF in South America are related to *Alouatta* [63–65].

Latitude and tropical ecosystem were not included in the final model; however our descriptive results serve as a good basis for the characterization of the geographic suitability of YF at continental level. In this study, YF-positive counties were located two degrees closer to the Equator, mostly within tropical ecosystems (78.6%), which are dominated by semi-evergreen and evergreen species, characterized by low variability in annual temperature and high levels of rainfall (>200 centimeter annually) [42]. Space-time analysis by county, locality or individual could help to better understand the dynamics enclosed in the tropical biomes.

Even though this study was not able to find an association between indicators of human activity, such as tree canopy loss and land use intensiveness (proxy for agriculture frontier), and risk of YF, further studies with a finer-scale approach are needed using other possible anthropogenic risk factors, such as deforestation, urbanization and population movement that are less noticeable in this geographic scale [66].

One of the limitations of this study was that it was not possible to find disaggregated information about YF vaccination coverage by county (study unit of analysis) for the 13 endemic countries during this 15-year study period. In the Americas, most countries with endemic areas have introduced the YF inoculation into their vaccination schedules as part of the Expanded Immunization Program [67]. Brazil’s immunization policy with respect to YF calls for vaccination after six months of age for people residing in transition zones and traveling to endemic areas [68]. There are several studies demonstrating the impact of mass vaccination in the reduction of YF cases [10, 11]. In Peru, a massive vaccination campaign was initiated in 2004 covering the endemic departments and areas where workers travel to the jungle during seasonal harvest and planting; a 90% coverage was achieved [68]. Nevertheless, while reviewing Table 1 of this study we can observe that the number of YF cases decreased only in 2007; since the location of where the vaccinations campaign took place was not available it was not possible to compare this information with the YF cases in the following period. In order for standardized information to be comparable between longer time periods, we recommend that future studies including vaccination coverages at subnational level are conducted by the countries, since they have information about the vaccination target population, the criteria for the selection of the target population and its coverage.

Immunization for residents of risk areas as well as for individuals involved in travel and commercial interchange in YF risk areas is imperative [36, 69]. All travelers to countries in which YF is endemic should be advised about the risk of the diseases and the prevention methods (personal protection and vaccine); as well about the possible adverse effects that may occur after vaccination [70]. Most of the YF cases reported in the Region of the Americas are related to agriculture workers [26, 71, 72]. Consequently, even if the endemic counties have good vaccination coverage, the movement of unvaccinated people to endemic areas by migration and tourism can represent a risk of new cases and possible outbreaks.

Another possible limitation of this paper is that ecological studies are commonly associated with the ecological fallacy, a possible erroneous inference that may occur because an association observed between variables on an aggregate level does not necessary represent or reflect the association that exists at individual level [73]. However, ecological type studies provide an inexpensive method of aggregating and comparing available data from countries’ surveillance systems and informing decision makers. In South America, the number of YF cases officially reported rely on passive surveillance and can be significantly underestimated [2]. In most South American endemic countries YF is a disease of compulsory notification, which is periodically published in the country’s Epidemiological Bulletins and yearly reported to PAHO/WHO as part of the International Health Regulations [74–78]. Nevertheless, taking into consideration this possible limitation, this study provides an important contribution by sharing the confirmed YF cases officially reported to PAHO from the 13 endemic countries of the Americas in the past 15 years.

In 2012, a group of YF specialists met in Panama to review the disease situation in the Americas in order to improve preparedness and response in terms of epidemiological, epizootic, entomological and laboratory surveillance [14]. As result, a series of recommendations were made based on existing data on the presence of either YFV or of YFV antibodies in humans, nonhuman primates or mosquitoes, with a view to better categorize the extent of the YF risk potential. Mapping, including standardized measurements of the geographical and epidemiological factors, was considered among the main recommendations.

Are the Americas at risk for urban YF outbreaks? The existing high density of *Aedes aegypti* in many urban areas of Latin America increase the risk of vector-borne diseases in region, demonstrated by outbreaks of dengue and chikungunya. Several studies estimate that vector-borne diseases will increase with climate change [79–81]. However, even if there are suitable environmental conditions and low vaccination rates that could represent a risk for the disease, epidemics, similar to the ones occurred in previous centuries, may not occur due the availability of vaccines to promptly stop urban transmission. The decision to increase vaccination coverage in risk areas is fundamental to protect this population from an outbreak.

The results of this study revealed that YF human cases in the Americas were reported in approximately 3% of the total number of counties in the region, mostly concentrated in three countries: Peru, Brazil and Colombia. This information can be used by decision-makers to allocate efforts and resources to specific areas. However, neighboring counties with no reported cases that share the same geo-environmental factors are at risk and need to be better surveyed.

In the spatial pattern analysis conducted in this study, we observed that several YF-positive counties were clustered, but there is always the risk of sporadic expansion towards neighboring areas that share similar ecological conditions with fewer cases or no cases reported. The 2016/2017 YF outbreak in Brazil is a recent example of how YF could emerge [18]. It began in the well-known endemic area of Minas Gerais, in the upper basin of the Doce River, which is characterized by a tropical forest habitat known locally as Bahia Interior forest, where YF clusters where detected in preceding years. The reported epizootics and human cases spread towards the Atlantic coast following the Doce River basin towards the state of Espirito Santo, outside the delimited endemic area. From there the cases extended over the local ecoregion known as Bahia Coastal Forest where there was not a routine vaccination program. Subsequently the outbreak radiated over the contiguous endemic areas of Minas Gerais where the tropical ecoregion Cerrado is predominant. Afterwards, YF expanded in the direction of the neighboring states of Sao Paulo and Rio de Janeiro, also a tropical ecosystem in the Paraná-Paraiba interior Forest and Serra do Mar Coastal Forest. These areas were not defined previously as endemic, and no YFV immunization was required.

Tropical and subtropical broadleaf forest seems to be the common denominator in the expansion of this recent outbreak. Few epizootics or human cases have been reported inside dryer areas. Perhaps there were fluctuations of temperature and rain within the tropical habitats that altered the natural cycles and created propitious circumstances for the spread of the virus, sickening NHP and humans in the area. It is very helpful to understand the geo-environmental conditions of these areas to predict where the YF epizootics and human cases can spread.

The 2016 outbreak reported in Angola, which spread cases to other countries within Africa and as far as China [12], suggests that many countries in our current globalized world are at risk for YF and other emerging diseases. During this outbreak, a worker returned to China from Africa with YF [82]; however, due to low temperatures in China and the absence of urban vectors, the spread of yellow fever to Asia, a region without circulation of this virus, was contained. A publication about the 2015–2016 YF outbreak in Angola, suggests that the extremely rapid unplanned urban migration in Africa by non-immune rural populations to already densely populated cities, where high densities of mosquitoes co-exist with city dwellers, has the potential for an epidemic of massive proportion in which political will combined with immunization is necessary [83].

Geospatial analysis and mapping are useful tools to detect and locate public health/disease spatial patterns and associated factors [84, 85]. This offers innovative possibilities of linking public health data to potential sources of environmental exposure [86]. Geographically processing environmental and epidemiological records allow the creation of a standardized and detailed digital database to spatially overlay and correlate environmental, socioeconomic and health data. It also allows the use of information from other sources/sectors and helps to gather and visualize statistics. This way, decision makers have a more inclusive set of elements to evaluate, delineate and focus efforts, and researchers can generate questions and hypothesis for future and more detailed studies.

Surveillance of arbovirus vectors of dengue, chikungunya and Zika viruses as well as their geographic determinants is essential for public health planning. Several modeling studies on vector global distribution suitability and risk mapping have been developed lately, including factors such as vegetation, land surface temperature, annual maximum and minimum precipitation [87–90], as well as anomalies and climatic variations, or cyclic events as El Niño or La Niña [91].

Countries’ available surveillance systems that have information about YF human cases and other arboviral infections can help us understand the spatial pattern of diseases and their related environmental factors in the region. An integrated approach for the surveillance, prevention and control of arboviral diseases was recommended by PAHO to the 55th Directing Council [92]. Yellow fever is an excellent example for the “One Health” approach, where the relationship between humans, animals and ecosystems need to be studied to improve knowledge on a disease and to enhance collaborative intersectoral and multidisciplinary control strategies. This is where geographic perspective (i.e. health geography, medical geography, geography of disease) improves the aforementioned approach of how to study the interaction between environmental dimensions and public health to identify and analyze time-space patterns of disease over the Earth’s surface [85, 93].

## Supporting information

S1 File Digital cartography data sources. (DOCX)

S2 File Influence of country level in the analysis. (DOCX)

S3 File Neighbors. (DOCX)

S4 File Example of a YF-positive county. (DOCX)

S5 File Distribution of YF cases by month and latitude within countries. (DOCX)

S6 File STROBE checklist. (DOCX)

## References

[1] World Health Organization. WHO Director-General summarizes the outcome of the Emergency Committee regarding clusters of microcephaly and Guillain-Barré syndrome [Internet]. Geneva: WHO; 2016 [cited 2016 September 15]. http://www.who.int/mediacentre/news/statements/2016/emergency-committee-zika-microcephaly/en/.

[2] Monath TP, Vasconcelos PF. Yellow fever. Journal of Clinical Virology. 2015;64:160–73. 10.1016/j.jcv.2014.08.030. 25453327

[3] Meade MS, Earickson RJ. Trasmissible disease systems -Yellow Fever In:. Medical geography. Second ed New York, The Guilford Press; 2000; p 61–70.

[4] Rogers DJ, Wilson AJ, Hay SI, Graham AJ. The Global Distribution of Yellow Fever and Dengue. Advances in parasitology. 2006;62:181–220. 10.1016/S0065-308X(05)62006-4 16647971 PMC3164798

[5] Bryant JE, Holmes EC, Barrett AD. Out of Africa: a molecular perspective on the introduction of yellow fever virus into the Americas. PLoS Pathog. 2007;3(5):e75 10.1371/journal.ppat.0030075 17511518 PMC1868956

[6] Gatrell AC, Elliott SJ. Explaining Geographies of Health In: Gatrell AC, Elliott SJ, editors. Geographies of health: An introduction. Third ed Sussex, UK: John Wiley & Sons; 2015 p. 25–49.

[7] Roig C, Miret J, Rojas A, Guillén Y, Aria L, Mendoza L, et al Estudio de Fiebre Amarilla en primates en áreas de brote de los departamentos de San Pedro y Central del Paraguay. Mem Inst Invest Cienc Salud (Impr). 2009;5(1):41–5.

[8] Acha P, Szyfres B. Zoonoses and communicable diseases common to man and animals: Chlamydiosis, Rickettsioses and Viroses. 3 ed Washington, D.C: Pan American Health Organization; 2003.

[9] Vasconcelos PF. [Yellow Fever]. Rev Soc Bras Med Trop. 2003;36(2):275–93. 12806465 10.1590/s0037-86822003000200012

[10] Garske T, Van Kerkhove MD, Yactayo S, Ronveaux O, Lewis RF, Staples JE, et al Yellow Fever in Africa: estimating the burden of disease and impact of mass vaccination from outbreak and serological data. PLoS Med. 2014;11(5):e1001638 10.1371/journal.pmed.1001638 24800812 PMC4011853

[11] Barnett ED. Yellow fever: epidemiology and prevention. Clinical infectious diseases: an official publication of the Infectious Diseases Society of America. 2007;44(6):850–6. 10.1086/511869 17304460

[12] World Health Organization. Yellow fever situation reports [Internet]. Geneva: WHO; 2016 [cited 2016 August 05]. http://www.who.int/emergencies/yellow-fever/situation-reports/archive/en/.

[13] World Health Organization. Risk assessment on yellow fever virus circulation in endemic countries: working document from an informal consultation of experts: a protocol risk assessment at the field level. Geneva: WHO, 2014.

[14] Pan American Health Organization. Technical Report: Recommendations for Scientific Evidence-Based Yellow Fever Risk Assessment in the Americas. Washington, DC: PAHO, 2013.

[15] World Health Organization. Background for the Consultation on Yellow Fever and International Travel, 2010 (update February 2011) Stockholm: WHO, 2011.

[16] Monath TP. Yellow fever. Medicine. 2005;33(7):21–3. 10.1383/medc.2005.33.7.21.

[17] Pan American Health Organization. Yellow Fever. Statistics and Maps. Number of cases and deaths [Internet]. Washington, DC: PAHO; 2012 [cited 2016 September 15]. http://www.paho.org/hq/index.php?option=com_content&view=article&id=8866&Itemid=40022&lang=en

[18] Pan American Health Organization/World Health Organization. Yellow fever: Epidemiological Alerts and Updates [Internet]. Washington, DC: PAHO/WHO; 2015 [cited 2016 December 01]. http://www.paho.org/hq/index.php?option=com_docman&task=doc_download&Itemid=270&gid=32648&lang=en

[19] May JM. The Ecology of Human Disease. New York: MD Publications Inc; 1959 351 p.

[20] Oppong J, Hamrold A. Disease, Ecology and Environment In: Brown T, McLafferty S, Moon G, editors. A companion to health and medical geography. West Sussex, UK: John Wiley & Sons; 2010 p. 81–95.

[21] Kumm HW, Cerqueira NL. The Haemagogus mosquitos of Brazil. Bulletin of Entomological Research. 1951;42(01):169–81.

[22] World Health Organization. International travel and health Chapter 6: Vaccine-preventable diseases and vaccines. Geneva: WHO, 2015.

[23] Johansson MA, Arana-Vizcarrondo N, Biggerstaff BJ, Staples JE. Incubation periods of Yellow fever virus. Am J Trop Med Hyg. 2010;83(1):183–8. 10.4269/ajtmh.2010.09-0782 20595499 PMC2912597

[24] Davis NC. The Effect of Various Temperatures in modifying the Extrinsic Incubation Period of the Yellow Fever Virus in Aedes aegypti. American Journal of Hygiene. 1932;16(1):163–76.

[25] Clements A. The biology of mosquitoes Volume 3, Viral, Arboviral and Bacterial Pathogens. Cambridge, MA: CABI; 2011 592 p.

[26] Vasconcelos PFC, Costa ZG, Travassos da Rosa ES, Luna E, Rodrigues SG, Barros VLRS, et al Epidemic of jungle yellow fever in Brazil, 2000: Implications of climatic alterations in disease spread. Journal of Medical Virology. 2001;65(3):598–604. 10.1002/jmv.2078 11596099

[27] Kelly HA. Walter Reed and yellow fever: McClure, Phillips; 1907.

[28] MacArthur RH. Geographical ecology: patterns in the distribution of species: Princeton University Press; 1972.

[29] Bensabath G, Shope RE, Andrade AHPd, Souza APd. Recuperación de virus amarílico, procedente de un mono centinela, en las cercanias de Belem, Brasil. Boletín de la Oficina Sanitaria Panamericana 1966;60(3):187–92. 4222266

[30] Davis NC. Susceptibility of Capuchin (Cebus) Monkeys to Yellow Fever Virus. American Journal of Hygiene. 1930;11(2):321–34.

[31] Davis NC, Shannon RC. Studies on South American Yellow Fever III. Transmission of The Virus To Brazilian Monkeys Preliminary Observations The Journal of experimental medicine. 1929;50(1):81–5. 19869607 10.1084/jem.50.1.81 PMC2131602

[32] Wolfe ND, Kilbourn AM, Karesh WB, Rahman HA, Bosi EJ, Cropp BC, et al Sylvatic transmission of arboviruses among Bornean orangutans. Am J Trop Med Hyg. 2001;64(5–6):310–6. 11463123 10.4269/ajtmh.2001.64.310

[33] Lima MA, Romano-Lieber NS, Duarte AMRdC. Circulation of antibodies against yellow fever virus in a simian population in the area of Porto Primavera Hydroelectric Plant, São Paulo, Brazil. Rev Inst Med Trop Sao Paulo. 2010;52(1):11–6. 20305949 10.1590/s0036-46652010000100002

[34] World Health Organization. International Health Regulations (2005). 2 ed Geneva: WHO; 2008.

[35] Jentes ES, Poumerol G, Gershman MD, Hill DR, Lemarchand J, Lewis RF, et al The revised global yellow fever risk map and recommendations for vaccination, 2010: consensus of the Informal WHO Working Group on Geographic Risk for Yellow Fever. The Lancet Infectious Diseases. 2011;11(8):622–32. 10.1016/S1473-3099(11)70147-5. 21798462

[36] World Health Organization. International travel and health. Geneva: WHO, 2012.

[37] Eisen L, Eisen RJ. Using geographic information systems and decision support systems for the prediction, prevention, and control of vector-borne diseases. Annual review of entomology. 2011;56:41–61. 10.1146/annurev-ento-120709-144847 20868280

[38] Schneider MC, Najera P, Aldighieri S, Galan DI, Bertherat E, Ruiz A, et al Where Does Human Plague Still Persist in Latin America? PLOS Neglected Tropical Diseases. 2014;8(2):e2680 10.1371/journal.pntd.0002680 24516682 PMC3916238

[39] United Nations Geographic Information Working Group (UNGIWG). Core Geo-Database. [Internet]. [cited 2016 August 05]. http://www.ungiwg.org/coreDB.

[40] De Smith MJ, Goodchild MF, Longley P. Geospatial analysis: a comprehensive guide to principles, techniques and software tools. 5th ed Winchelsea, UK: The Winchelsea Press; 2015 60 p.

[41] U.S. Geological Survey (USGS). HYDRO1K [Internet]. US Geological Survey's Earth Resources Observation and Science (EROS) Center; 2015 [updated January 2015; cited 2016 September 15]. https://lta.cr.usgs.gov/HYDRO1K.

[42] World Wildlife Fund (WWF). Major Habitat Types [Internet]. [cited 2016 September 30]. http://wwf.panda.org/about_our_earth/ecoregions/about/habitat_types/.

[43] Hijmans RJ, Cameron SE, Parra JL, Jones PG, Jarvis A. Very high resolution interpolated climate surfaces for global land areas. International Journal of Climatology. 2005;25(15):1965–78. 10.1002/joc.1276

[44] WorldClim: Global Climate Data. Bioclimatic variables [cited 2016 September 30]. http://www.worldclim.org/bioclim.

[45] Nachtergaele F, Petri M. Mapping land use systems at global and regional scales for land degradation assessment analysis. Rome: FAO, 2008.

[46] FAO. GeoNetwork. Land Use systems of the World. Latin American and Caribbean [Internet]. 2008 [cited 2016 August 15]. http://www.fao.org/geonetwork/srv/en/main.home.

[47] Hansen MC, Potapov PV, Moore R, Hancher M, Turubanova SA, Tyukavina A, et al High-Resolution Global Maps of 21st-Century Forest Cover Change. Science. 2013;342(6160):850–3. 10.1126/science.1244693 24233722

[48] Hansen MC, Potapov PV, Moore R, Hancher M, Turubanova SA, Tyukavina A, et al. Global Forest Change [Internet]. [cited 2016 August 20]. http://earthenginepartners.appspot.com/science-2013-global-forest.

[49] International Union for Conservation of Nature and Natural Resources. Terrestrial mammals location database [Internet]. 2015 [cited 2016 October 10]. http://www.iucnredlist.org/initiatives/mammals.

[50] Pfeiffer D, Robinson T, Stevens K, Rogers D, Clements A. Spatial Analysis in Epidemiology. New York, NY: Oxford University Press; 2008 142 p.

[51] Robinson AH, Morrison JL, Muehrcke PC, Kimerling AJ, Guptill SC. Elements of Cartography. 6th ed New York: Wiley 1995 688 p.

[52] Esri: ArcGIS Pro. How inverse distance weighted interpolation works [Internet]. [cited 2016 September 15]. http://pro.arcgis.com/en/pro-app/help/analysis/geostatistical-analyst/how-inverse-distance-weighted-interpolation-works.htm.

[53] Dohoo IR, Martin W, Stryhn HE. Veterinary epidemiologic research. 2nd ed: VER Inc; 2009.

[54] Kumm HW, Osorno-Mesa E, Boshell-Manrique J. Studies on Mosquitoes of the Genus Haemagogus in Colombia (Diptera, Culieidae). American journal of hygiene. 1946;43(1):13–28. 21011557 10.1093/oxfordjournals.aje.a119048

[55] Kottek M, Grieser J, Beck C, Rudolf B, Rubel F. World Map of the Köppen-Geiger climate classification updated. Meteorologische Zeitschrift. 2006;15(3):259–63. 10.1127/0941-2948/2006/0130

[56] Matsuura K, Willmott C. South America rainfall climatology [Internet]. 2001 [cited 2015 October 01]. http://research.jisao.washington.edu/data_sets/ud/samerica/.

[57] Chadee DD, Tikasingh ES, Ganesh R. Seasonality, biting cycle and parity of the yellow fever vector mosquito Haemagogus janthinomys in Trinidad. Medical and veterinary entomology. 1992;6(2):143–8. 1358266 10.1111/j.1365-2915.1992.tb00592.x

[58] Tubaki RM, Menezes RM, Vesgueiro FT, Cardoso RP Jr. Observations on Haemagogus janthinomys Dyar (Diptera: Culicidae) and other mosquito populations within tree holes in a gallery forest in the northwestern region of Sao Paulo state, Brazil. Neotropical entomology. 2010;39(4):664–70. 20878007 10.1590/s1519-566x2010000400030

[59] Pinto CS, Confalonieri UE, Mascarenhas BM. Ecology of Haemagogus sp. and Sabethes sp. (Diptera: Culicidae) in relation to the microclimates of the Caxiuanã National Forest, Pará, Brazil. Memórias do Instituto Oswaldo Cruz. 2009;104:592–8. 19722082 10.1590/s0074-02762009000400010

[60] Hindle E. The Transmission of Yellow Fever. The Lancet. 1930;216(5590):835–42.

[61] Christophers S. Aedes aegypti (L.) the yellow fever mosquito: its life history, bionomics and structure. New York: Cambridge University Press; 1960 751 p.

[62] Moreno ES, Barata Rde C. Methodology for definition of yellow fever priority areas, based on environmental variables and multiple correspondence analyses. PLoS Negl Trop Dis. 2012;6(7):e1658 10.1371/journal.pntd.0001658 22802971 PMC3389021

[63] Almeida MA, Cardoso JdC, dos Santos E, da Fonseca DF, Cruz LL, Faraco FJ, et al Surveillance for yellow fever virus in non-human primates in Southern Brazil, 2001–2011: A tool for prioritizing human populations for vaccination. PLoS Negl Trop Dis. 2014;8(3):e2741 10.1371/journal.pntd.0002741 24625681 PMC3953010

[64] Almeida MABd, Santos Ed, Cardoso JdC, Fonseca DFd, Noll CA, Silveira VR, et al Yellow fever outbreak affecting Alouatta populations in southern Brazil (Rio Grande do Sul State), 2008–2009. American Journal of Primatology. 2012;74(1):68–76. 10.1002/ajp.21010 22020690

[65] Holzmann I, Agostini I, Areta JI, Ferreyra H, Beldomenico P, Di Bitetti MS. Impact of yellow fever outbreaks on two howler monkey species (Alouatta guariba clamitans and A. caraya) in Misiones, Argentina. American Journal of Primatology. 2010;72(6):475–80. 10.1002/ajp.20796 20095025

[66] Rican S, Salem G. Mapping Disease In: Brown T, McLafferty S, Moon G, editors. A companion to health and medical geography. West Sussex, UK: John Wiley & Sons; 2010 p. 96–110.

[67] World Health Organization. Expanded Programme on Immunization: Report of the 13th Global Advisory Group Meeting. Cairo, Egypt: WHO, 1990.

[68] PAHO. Health in the Americas. Washington D.C.: Pan American Health Organization; 2007 756 p.

[69] Monath TP. Yellow fever: an update. The Lancet Infectious diseases. 2001;1(1):11–20. 10.1016/S1473-3099(01)00016-0 11871403

[70] Staples JE, Gershman M, Fischer M. Yellow fever vaccine: recommendations of the Advisory Committee on Immunization Practices (ACIP). Morbidity and Mortality Weekly Report. 2010;59(RR07):1–27. 20671663

[71] PAHO. Health in the Americas. Washington D.C.: Pan American Health Organization; 2007 454 p.

[72] Câmara FP, de Carvalho LM, Gomes ALB. Demographic profile of sylvatic yellow fever in Brazil from 1973 to 2008. Transactions of The Royal Society of Tropical Medicine and Hygiene. 2013;107(5):324–7. 10.1093/trstmh/trt014 23442573

[73] Porta M. A dictionary of epidemiology: Oxford University Press; 2008.

[74] Pan American Health Organization. Detection, Verification and Risk Assessment (DVA) [Internet]. Washington, DC: PAHO; [updated 2017 June 01; cited 2017 June 05].

[75] Ministry of Health of Brazil. Epidemiological situation in Brazil [Internet]. Brasilia, Brazil: Secretary of health; 2016 [cited 2017 June 07]. http://portalsaude.saude.gov.br/index.php/situacao-epidemiologica-dados-febreamarela.

[76] Ministry of Health of Colombia. Epidemiological Bulletin, 28 May—03 June [Internet]. Bogota, Colombia: National Institute of Health of Colombia; 2017 [cited 2017 June 07]. http://www.ins.gov.co/boletin-epidemiologico/Boletn%20Epidemiolgico/2017%20Bolet%C3%ADn%20epidemiol%C3%B3gico%20semana%2022.pdf.

[77] Ministry of Health of Ecuador. Epidemiological Bulletin [Internet]. Quito, Ecuador: National Secretary of Public Health Surveillance; 2017 [updated 2017 May 05; cited 2017 June 07]. http://www.salud.gob.ec/wp-content/uploads/downloads/2017/06/5-FA_SE-21.pdf.

[78] Ministry of Health of Peru. Epidemiological Bulletin of Peru, 23–29 Abril 2017 [Internet]. 2017 [cited 2017 June 07]. http://www.dge.gob.pe/portal/docs/vigilancia/boletines/2017/17.pdf.

[79] Patz JA, Campbell-Lendrum D, Holloway T, Foley JA. Impact of regional climate change on human health. Nature. 2005;438(7066):310–7. Epub 2005/11/18. 10.1038/nature04188 16292302

[80] IPCC. Climate Change 2007: Impacts, Adaptation and Vulnerability. Contribution of Working Group II to the Fourth Assessment Report of the Intergovernmental Panel on Climate Change. Cambridge, UK: Intergovernmental Panel on Climate Change, 2007.

[81] PAHO. Climate change and human health: risk and responses: revised summary 2008 Washinton, DC: Pan American Health Organization, 2008.

[82] Chen Z, Liu L, Lv Y, Zhang W, Li J, Zhang Y, et al A fatal yellow fever virus infection in China: description and lessons. Emerging Microbes and Infections. 2016;5(7):e69 10.1038/emi.2016.89 27406389 PMC5141266

[83] Ahmed QA, Memish ZA. Yellow fever from Angola and Congo: a storm gathers. Tropical Doctor. 2017;47(2):92–6. 10.1177/0049475517699726 28424031

[84] Beale L. Effective use of GIS for spatial epidemiology In: Kanaroglou P, Delmelle E, Paez A, editors. Spatial analysis in health geography. Surrey, England: Ashgate Publishing, Ltd; 2015 p. 15–30.

[85] Delmelle E, Kanaroglou P. Introduction: Spatial Analysis and Health In: Kanaroglou P, Delmelle E, Paez A, editors. Spatial analysis in health geography. Surrey, England: Ashgate Publishing, Ltd; 2015 p. 1–14.

[86] Rushton G. Public Health, GIS, and Spatial Analytic Tools. Annual Review of Public Health. 2003;24(1):43. 10.1146/annurev.publhealth.24.012902.140843 12471269

[87] Guzman MG, Halstead SB, Artsob H, Buchy P, Farrar J, Gubler DJ, et al Dengue: a continuing global threat. Nature Reviews Microbiology. 2010;8:S7–S16. 10.1038/nrmicro2460 21079655 PMC4333201

[88] Kraemer MUG, Sinka ME, Duda KA, Mylne AQN, Shearer FM, Barker CM, et al The global distribution of the arbovirus vectors Aedes aegypti and Ae. albopictus. eLife. 2015;4:e08347 10.7554/eLife.08347 26126267 PMC4493616

[89] Nsoesie EO, Kraemer MU, Golding N, Pigott DM, Brady OJ, Moyes CL, et al Global distribution and environmental suitability for chikungunya virus, 1952 to 2015. Euro surveillance: European communicable disease bulletin. 2016;21(20):1–12. 10.2807/1560-7917.ES.2016.21.20.30234. PMC4902126 27239817

[90] Messina JP, Kraemer MUG, Brady OJ, Pigott DM, Shearer FM, Weiss DJ, et al Mapping global environmental suitability for Zika virus. eLife. 2016;5:e15272 10.7554/eLife.15272 27090089 PMC4889326

[91] Munoz AG TM, Goddard LM., Aldighieri S. The Latin American and Caribbean Climate Landscape for ZIKV Transmission. IRI Technical Report: Columbia University Academic Commons, 2016.

[92] Pan American Health Organization. Strategy for Arboviral Disease Prevention and Control. Resolution CD55.R6. 55th Directing Council. Washington, DC: PAHO, 2016.

[93] Mayer J. Medical geography In: Brown T, McLafferty S, Moon G, editors. A companion to health and medical geography. West Sussex, UK: John Wiley & Sons; 2010 p. 33–54.

