# Supplementary material for: Geographic patterns and environmental factors associated with human yellow fever presence in the Americas
Source: PLoS Negl Trop Dis. 2017 Sep 8;11(9):e0005897. doi: 10.1371/journal.pntd.0005897 (PMC5607216; doi:10.1371/journal.pntd.0005897)
Supplement: S1 File — (DOCX) [file pntd.0005897.s001.docx]

# S1 File. Digital cartography data sources

| Variable | Measurement / Indicator | Source |
| --- | --- | --- |
| Latitude | Geographic latitude and longitude measured in decimal degrees from the polygon centroid. | UNGIWG-SALB-PAHO 2007-2013: <http://www.unsalb.org/> |
| Minimum Altitude | Lowest altitude measured within the county polygon in meters above sea level.  Classified using natural breaks: 0-317; 318-784; 785-1,808; > 1,809 | USGS-HYDRO1K 2006Digital Elevation Model –DEM-, 2006 courtesy of the U.S. Geological Survey. Original GTOPO30US Geological Survey's Earth Resources Observation and Science (EROS) Center <https://lta.cr.usgs.gov/HYDRO1K> |
| Hydrology | Used as reference on maps.  Classified by the *Strahler* order of the watercourse considering the number of tributaries of each stream segment | USGS-HYDRO1K 2006Stream LinesUS Geological Survey's Earth Resources Observation and Science (EROS) Center<https://lta.cr.usgs.gov/HYDRO1K> |
| Major Habitat Type (MHT) | Calculated using overlapping geoprocessing function and obtaining the county percentage covered by each MHT.  The original 11 categories were reclassified as:  **Tropical**: Tropical and subtropical moist broadleaf forests, Tropical and subtropical grasslands, savannas, and shrublands, Tropical and subtropical dry broadleaf forests, mangroves, **No-Tropical**: Temperate coniferous forests, Temperate broadleaf and mixed forests, Montane grasslands, Mediterranean scrub, Flooded grasslands, Deserts and xeric shrublands | WWF Global - Major Habitat Types: <http://wwf.panda.org/about_our_earth/ecoregions/about/habitat_types/>  FAO-GeoNetwork-geo-spatial - World Wild Fund Global Ecoregions Map [Internet]. [cited 2014 Dec 10] <http://www.fao.org/geonetwork/srv/en/resources.get?id=1009&fname=1009.zip&access=private>  Or directly from UNEP 2014 (United Nations Environment Programme) <http://ede.grid.unep.ch//mod_download/download_geospatial.php?selectedID=1814&newFile=download/wwf_ecoreg_tot_po_shp.zip> |
| Temperature | Annual Mean Temperature (WorldClim BIO1). Measured in Celsius scale and geoprocessed by county  Classified using natural breaks: 3.0 - 14.3; 14.4 - 20.0; 20.1 - 23.9; 24.0 - 28.7 | WorldClim - Global Climate Data. BIOCLIM-Bioclimatic variables. Hijmans, R.J., S.E. Cameron, J.L. Parra, P.G. Jones and A. Jarvis, 2005. Very high resolution interpolated climate surfaces for global land areas. International Journal of Climatology 25: 1965-1978. [http://www.worldclim.org/bioclimhttp://www.worldclim.org/bioclim](http://www.worldclim.org/bioclim) |
| Precipitation (rain) | Annual Precipitation (WorldClim BIO12)  Measured in millimeters and geoprocessed by county  Classified using natural breaks: 3 – 1,066; 1,067 - 1,722; 1,723 - 2,762; 2,763 - 8,233 | WorldClim - Global Climate Data . BIOCLIM-Bioclimatic variables. Hijmans, R.J., S.E. Cameron, J.L. Parra, P.G. Jones and A. Jarvis, 2005. Very high resolution interpolated climate surfaces for global land areas. International Journal of Climatology 25: 1965-1978. [http://www.worldclim.org/bioclimhttp://www.worldclim.org/bioclim](http://www.worldclim.org/bioclim) |
| Non-Human Primate (NHP) Hosts | Calculated using overlapping geoprocessing function and obtaining the presence of total hosts per county (Alouatta, Aotus, Callithrix, Saguinus, Ateles, Cebus, Saimiri, Lagothrix) | IUCN 2015  The IUCN Red list maps with the species as sub-species location of terrestrial mammals’ database of these primates <http://www.iucnredlist.org/initiatives/mammals>  Spatial data download  <http://www.iucnredlist.org/technical-documents/spatial-data> |
| Canopy Tree disruption or Loss | Calculated using overlapping geoprocessing function and obtaining the county percentage covered with tree canopy loss > 30% (2000-2012) | Hansen, M. C., P. V. Potapov, R. Moore, M. Hancher, S. A. Turubanova, A. Tyukavina, D. Thau, S. V. Stehman, S. J. Goetz, T. R. Loveland, A. Kommareddy, A. Egorov, L. Chini, C. O. Justice, and J. R. G. Townshend. 2013. “High-Resolution Global Maps of 21st-Century Forest Cover Change.” Science 342 (15 November): 850–53. Data available on-line from: <http://earthenginepartners.appspot.com/science-2013-global-forest>. |
| **Frontier**  (Land use intensiveness) | Calculated using overlapping geoprocessing function named Spatial Statistics\Majority  The original 40 categories were reclassified as following combinations of land use and intensiveness:  In “Frontier” were included counties with high to moderate use of natural lands (as forest, shrubs, grassland, wetlands)  Not included in “Frontier**”** were urban areas and high-to-moderate land use of crops-agricultural zones, bare areas; as well as low-use of natural lands (i.e. forest, shrubs, grassland, sparse vegetation, or wetlands) | LUS 2010  Land Use systems of the World. Latin American & Caribbean, 2008  LADA 2008 Mapping Land Use Systems at global and regional scales for Land Degradation Assessment Analysis. Nachtergaele F & Petri M. LADA Technical report n.8, version 1.1 Available in FAO Geonetwork, [http://www.fao.org/geonetwork/srv/en/main.homehttp://www.fao.org/geonetwork/srv/en/main.home](http://www.fao.org/geonetwork/srv/en/main.home)  Online resource data for download: Land use systems ESRI GRID (Rev 2010). <http://www.fao.org/geonetwork/srv/en/resources.get?id=37139&fname=lus.zip&access=private> Downloaded in January 2012. |
