# Supplementary material for: Geographic patterns and environmental factors associated with human yellow fever presence in the Americas
Source: PLoS Negl Trop Dis. 2017 Sep 8;11(9):e0005897. doi: 10.1371/journal.pntd.0005897 (PMC5607216; doi:10.1371/journal.pntd.0005897)
Supplement: S2 File — (DOCX) [file pntd.0005897.s002.docx]

# S2 File. Influence of country level in the analysis.

Table. Factors contributing to the probability of yellow fever human cases presence per county (yellow fever positive county) (P<0.15 in the bivariate and multivariate analysis and P<0.05), 2000-2014- Countries

| **Variables** | **No.(%)*/Median (IQR)** | ***P*-Value** | **Odds Ratio (CI _95%_)** |
| --- | --- | --- | --- |
| **Country** |  |  |  |
| **Argentina** | 511 (6.03%) | - | - |
| **Bolivia** | 112 (1.3%) | < 0.001 | 1.23 (1.19-1.28) |
| **Brazil** | 5,564 (65.7%) | 0.05 | 1.01 (0.99-1.03) |
| **Colombia** | 1,107 (13.1%) | <0.001 | 1.03 (1.01-1.05) |
| **Ecuador** | 221 (2.6%) | 0.95 | 0.99 (0.97-1.02) |
| **French Guiana** | 22 (0.3%) | 0.79 | 0.99 (0.91-1.06) |
| **Guyana** | 26 (0.3%) | 0.78 | 0.99 (0.92-1.06) |
| **Panama** | 79 (0.9%) | 0.64 | 0.99 (0.94-1.03) |
| **Paraguay** | 217 (2.6%) | 0.21 | 1.01 (0.98-1.04) |
| **Peru** | 194 (2.3%) | <0.001 | 1.21 (1.18-1.25) |
| **Suriname** | 62 (0.7%) | 0.68 | 0.99 (0.94-1.08) |
| **Trinidad and Tobago** | 15 (0.2%) | 0.83 | 0.99 (0.90-1.08) |
| **Venezuela** | 335 (4%) | 0.001 | 1.04 (1.01-1.06) |

Analyzing by country and having Argentina as a reference, the probability of finding positive YF counties was **1.23** times in Bolivia (p< 0.001; CI_95%_=1.19-1.28), **1.21** times higher in Peru (p< 0.001; CI_95%_=1.18-1.25), **1.04** times in Venezuela (p= 0.001; CI_95%_= 1.01-1.06), **1.03** times in Colombia (p<0.001; CI_95%_=1.01-1.05).

Ecuador and Paraguay had few cases during the study period but differences were not statistically significant, neither were the countries without reported YF cases during the study period: Panama, French Guiana, Guyana, Suriname and Trinidad & Tobago.

Additionally, and noting the number of cases concentrated in three countries and the spatial clusters found, we decided to ran an alternative multivariate mixed model in which country was the control variable. The Intra class correlation (ICC) for the country effect was measured on the outcome (YF cases). The results suggest that country has little effect on the outcome considering a mixed model approach. The ICC indicates that ~4% of the variance in 'YF cases' can be "explained" by country.
