# Supplementary material for: Geographic patterns and environmental factors associated with human yellow fever presence in the Americas
Source: PLoS Negl Trop Dis. 2017 Sep 8;11(9):e0005897. doi: 10.1371/journal.pntd.0005897 (PMC5607216; doi:10.1371/journal.pntd.0005897)
Supplement: S3 File — (DOCX) [file pntd.0005897.s003.docx]

# S3 File. Neighbors

Geographic proximity techniques were used to identify the productive areas’ contiguous neighbors. In order to identify counties which are neighbors of locations with YF cases, an ArcGIS spatial selection method was applied “select by location that touch the boundary of the sources layer feature”, being the source feature those areas with reported YF cases during the 2000-2014 period.

**Table 1. YF counties and neighbors comparison**

| Variable | YF (Not a neighbor 286) | YF (Neighbor 791) | Mann-Whitney U test |
| --- | --- | --- | --- |
|  | *Median  (1 quartile-3 quartile)* | *Median  (1 quartile-3 quartile)* |  |
| Latitude | -12.46 (-16.93- -1.00) | -14.20 (-19.21- -1.23) | P=0.007 |
| Altitude | 234.5 (89-459.5) | 298 (92.5-589.5) | P=0.001 |
| Temperature | 24.00 (21.52-25.78) | 23.40 (20.30-25.60) | P=0.01 |
| Precipitation | 1681 (1410-2221) | 1529 (1325-1874) | P<0.001 |
| Total host NHP | 4 (3-6) | 3 (2-5) | P<0.001 |
| Tropical | 1 (1-1) | 1 (1-1) | P =0.22 |
| Frontier | 0 (0-1) | 0 (0-1) | P=0.54 |

As we identified YF county neighbors using proximity techniques and then compared their geographic and environmental conditions, using Mann-Whitney U test, we found that neighbors are similar to YF counties in their tropical habitat and land use intensity (frontier).

YF counties and neighbors are different in terms of altitude, latitude, temperature, annual rainfall and number of total hosts. Neighbors registered higher southern latitude (almost 2 degrees) with a wider interquartile range; their altitude 63 meters higher; their annual precipitation amount is smaller (152 mm less) and they record fewer amounts of NHP hosts. Temperature is cooler in neighbors by a fraction of Celsius degree.
