# Supplementary material for: Geographic patterns and environmental factors associated with human yellow fever presence in the Americas
Source: PLoS Negl Trop Dis. 2017 Sep 8;11(9):e0005897. doi: 10.1371/journal.pntd.0005897 (PMC5607216; doi:10.1371/journal.pntd.0005897)
Supplement: S4 File — (DOCX) [file pntd.0005897.s004.docx]

**S4 File. Example of a YF positive county**

One area was selected to exemplify the profile of a typical YF positive county that fits the geo-environmental factors studied. The county (locally known as province) of **Chanchamayo** in the north of the Junin Region in central Peru (latitude: 11.03^o^S) reported 53 YF human cases throughout 9 out of the 15 years studied.


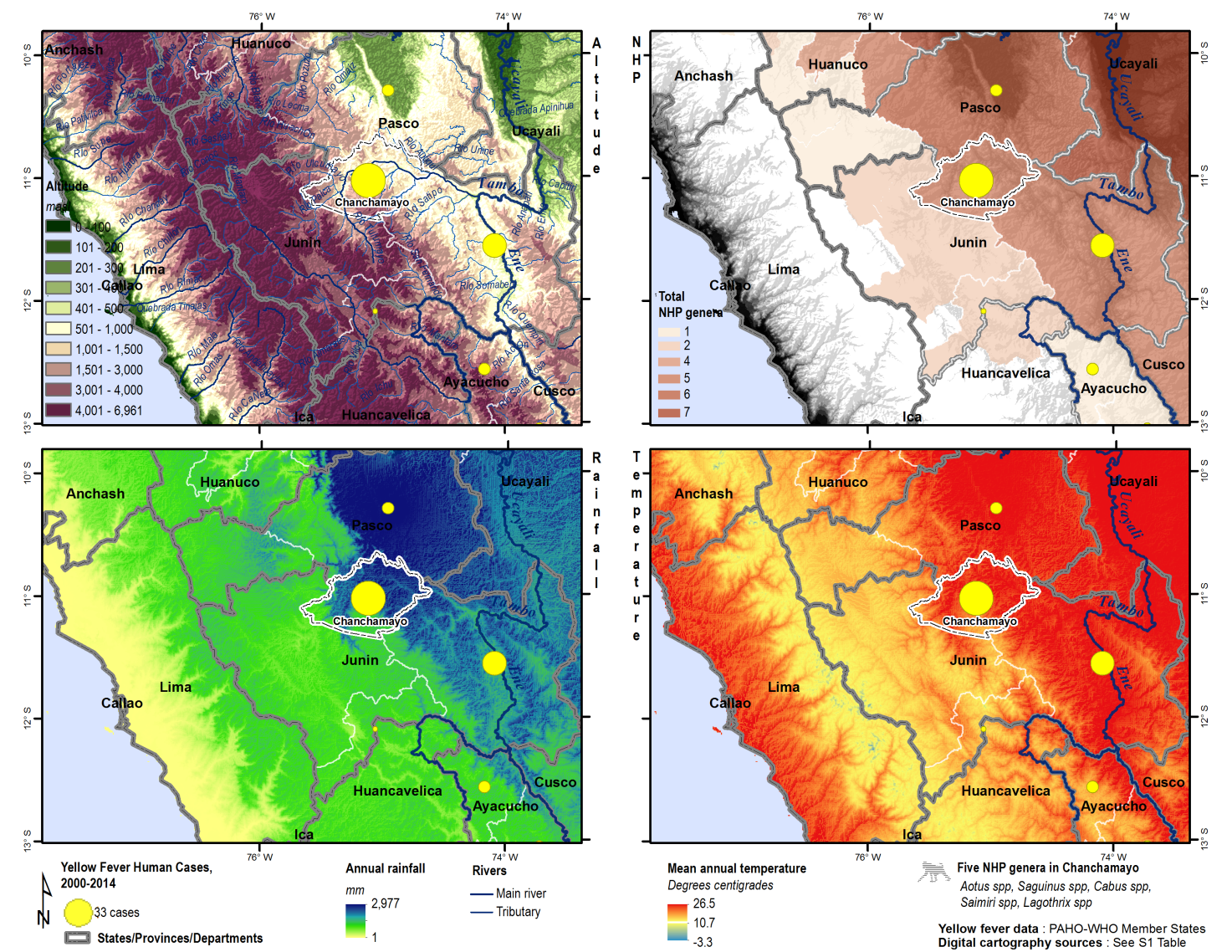
**Fig 7. Chanchamayo, Junín in Peru: a representative example of a YF positive county.**

This county is part of the YF geographic clusters along the Andes foothills. During 2004 it registered an outbreak with 33 cases and another 10 cases in the course of 2006.

Its lowest altitude is 432 meters above sea level is situated nearby the Chanchamayu River, flowing towards the Perené and Tambo tributaries of the Ucayali, and ending up at the confluence with the Marañon River on the Amazon upper basin.

The county belongs to the Peruvian Yungas, local instance of the South America Tropical and subtropical moist broadleaf forest; with a total 1,733 mm of annual precipitation and mean temperature of 18.6 o C. The presence of five different genera of NHP has been reported in the area: *Aotus*, *Saguinus*, *Cebus*, *Saimiri* and *Lagothrix*.

During 2016, Chanchamayo experienced newer outbreaks of YF, in addition to other arbovirus infections [1], indicating persistent geo-ecological conditions favouring the persistence of viral transmission.

**Reference**

1. Ministerio de Salud del Peru. Boletin Epidemiologico del Peru [Internet]. 2016 [cited 2016 December 15]. Available from: <http://www.dge.gob.pe/portal/docs/vigilancia/boletines/2016/45.pdf>
