# Supplementary material for: Geographic patterns and environmental factors associated with human yellow fever presence in the Americas
Source: PLoS Negl Trop Dis. 2017 Sep 8;11(9):e0005897. doi: 10.1371/journal.pntd.0005897 (PMC5607216; doi:10.1371/journal.pntd.0005897)
Supplement: S5 File — (DOCX) [file pntd.0005897.s005.docx]

**S5 File. Distribution of YF cases by month and latitude within countries**

The figure below presents the distribution of yellow fever cases by month of occurrence and location (country and first administrative division) according their position with regards to the Equator. Colombia and Venezuela, on the north, have reported more cases during June and July, Brazil and Paraguay reported more cases from December to March. Peru notifies 37.7% of the total cases in the region and have cases throughout the year, with 11.7% of the cases reported in December. This preliminary examination suggests that there are different periods of the year with higher number of cases reported, which may be related seasonality of climatic conditions that need to be deeper analyzed in future studies. Seasonal patterns of South American countries variate according with their latitudinal situation, as well as other climatic factors like continental mass and water body closeness, topography, wind patterns and ocean currents.

**Figure 8. Yellow fever cases by month, by country/first administrative level and by latitude, South America, 2000 to 2014**


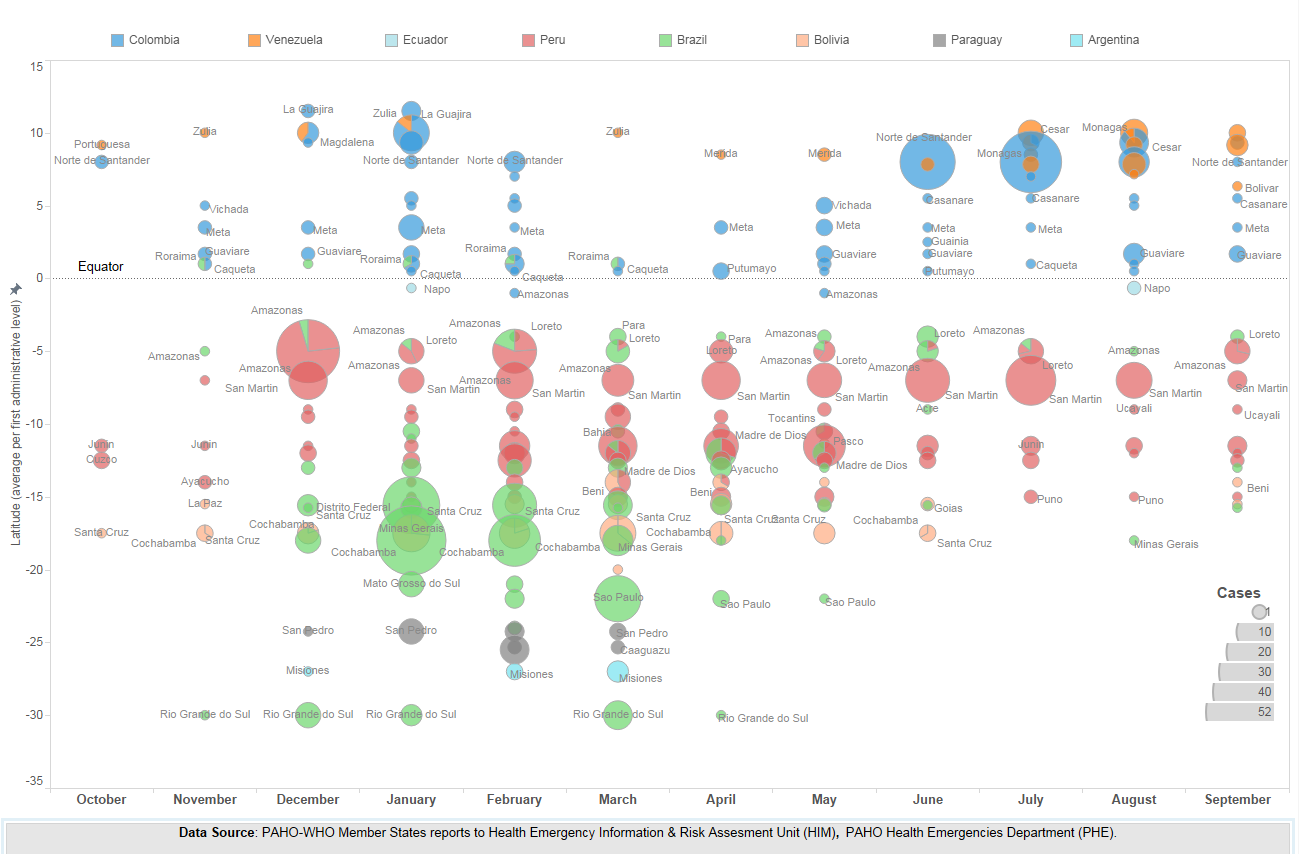


Source: Pan American Health Organization/ PAHO Health Emergencies Department (PHE). Available online at: <http://ais.paho.org/phip/viz/ed_yellowfever.asp>
